# Secondary structural choice of DNA and RNA associated with CGG/CCG trinucleotide repeat expansion rationalizes the RNA misprocessing in FXTAS

**DOI:** 10.1038/s41598-021-87097-y

**Published:** 2021-04-14

**Authors:** Yogeeshwar Ajjugal, Narendar Kolimi, Thenmalarchelvi Rathinavelan

**Affiliations:** Department of Biotechnology, Indian Institute of Technology Hyderabad, Kandi, Telangana State 502285 India

**Keywords:** Biophysics, Computational biology and bioinformatics, Structural biology

## Abstract

CGG tandem repeat expansion in the 5′-untranslated region of the *fragile X mental retardation-1* (*FMR1*) gene leads to unusual nucleic acid conformations, hence causing genetic instabilities. We show that the number of G…G (in CGG repeat) or C…C (in CCG repeat) mismatches (other than A…T, T…A, C…G and G…C canonical base pairs) dictates the secondary structural choice of the sense and antisense strands of the *FMR1* gene and their corresponding transcripts in fragile X-associated tremor/ataxia syndrome (FXTAS). The circular dichroism (CD) spectra and electrophoretic mobility shift assay (EMSA) reveal that CGG DNA (sense strand of the *FMR1* gene) and its transcript favor a quadruplex structure. CD, EMSA and molecular dynamics (MD) simulations also show that more than four C…C mismatches cannot be accommodated in the RNA duplex consisting of the CCG repeat (antisense transcript); instead, it favors an i-motif conformational intermediate. Such a preference for unusual secondary structures provides a convincing justification for the RNA foci formation due to the sequestration of RNA-binding proteins to the bidirectional transcripts and the repeat-associated non-AUG translation that are observed in FXTAS. The results presented here also suggest that small molecule modulators that can destabilize *FMR1* CGG DNA and RNA quadruplex structures could be promising candidates for treating FXTAS.

## Introduction

The eukaryotic genome comprises ubiquitous repetitive sequences, namely microsatellites. Although microsatellites can be tracts of repetitive nucleotides with the lengths varying between 1 and 6, certain trinucleotide microsatellite is prone to undergo expansion, resulting in a variety of genetic disorders^1–3^. Such a catastrophic DNA damage has consequences within many biological processes, such as replication, transcription, repair, and recombination processes, leading to several neurological disorders. When the number of trinucleotide repeats exceeds the threshold, it forms an unusual nucleic acid conformations^4^. One such example is CGG trinucleotide repeat expansion in the *fragile X mental retardation-1* (*FMR1*) gene.

*FMR1* gene encodes for fragile X mental retardation protein (FMRP), which is an RNA binding protein and is essential for the brain development^5^. The 5′-untranslated region (5′ UTR) of the *FMR1* gene has CGG tandem repeats, and when the repeats expand beyond 200, this leads to fragile X syndrome (FXS). The prevalence of FXS is approximately 1 in 4000 males and 1 in 8000 females, and it populates ~ 30% of all X-linked disorders^6^. Nonetheless, when the CGG repeat number lies between 55 and 200 in premutation carrier individuals, it leads to fragile X-associated tremor/ataxia syndrome (FXTAS)^7^. One in 150–300 and 400–850 women and men, respectively, in the general populations are found to be the carriers of the FMR1 premutation state^8,9^. Among the premutation carrier individuals, about 40–75% and 16–20% of males and females, respectively, develops FXTAS at an older age^10^.

Although the total inhibition of FMRP is seen in FXS^11^, complex RNA misprocessing mechanisms can be observed in FXTAS^12^. A bidirectional transcription of the *FMR1* gene and concomitant repeat-associated non-AUG translation (RAN) are among these misprocessing mechanisms. The unusual secondary structural choice of CGG (sense strand) and CCG (antisense strand) repeats at both the DNA and RNA levels could be traced to this RNA misprocessing. However, there are controversial evidences on the secondary structural preference of DNA and RNA strands consisting of CGG repeats^13–16^. Although some studies suggest a hairpin structure formation^14,17^, the others favor quadruplex formation^18^, wherein 4 guanines engaged in a Hoogsteen base pairing stack onto each other in a helix. Similarly, the complementary CCG repeat can favor a four-stranded i-motif structure^19^, wherein the cytosines are engaged in C+…C (at acidic pH) or C…C (at non-acidic pH) base pairing in an intercalating fashion. However, the secondary structural preference for CCG repeats in the context of a number of repeats remains elusive^20–22^. Coincidently, fragile XE syndrome (FRAXE), an X-linked disorder, is caused by the abnormal expansion of CCG triplet repeats that are present in the 5′ UTR of *FMR2* (also called, *AFF2)* gene^23–25^. The protein encoded by the *FMR2* gene acts as a transcription factor that is essential for the cognitive development^5^. The number of CCG repeats in the *FMR2* gene occurs between 60 (found in normal individuals) and 200 in the premutated state, whereas it occurs above 200 in the full mutated state^24–26^.

In the current study, we investigate the secondary structural choice of CCG and CGG repeats from the perspective of addressing the molecular basis of FXTAS by employing molecular dynamics (MD) simulations, circular dichroism (CD), and electrophoretic mobility shift assay (EMSA). The results show the preference for a quadruplex by both the CGG sense strand (DNA) and the sense transcript (RNA). Interestingly, although the antisense CCG strand favors the hairpin structure, the antisense transcript prefers the i-motif/i-motif conformational intermediate structure. Such a noncanonical secondary structural choice may be the underlying molecular cause for the RNA misprocessing in FXTAS. The mechanism proposed here, which is based on the secondary structural choice of CGG (quadruplex) and CCG (i-motif/i-motif conformational intermediate) repeats, explains the neurotoxicity observed in FXTAS.

## Results

MD, EMSA, and CD investigations have been carried out to explore the association between the repeat number and secondary structural preference for the DNA and RNA sequences consisting of the CGG and CCG repeats (Table 1).

**Table 1:**
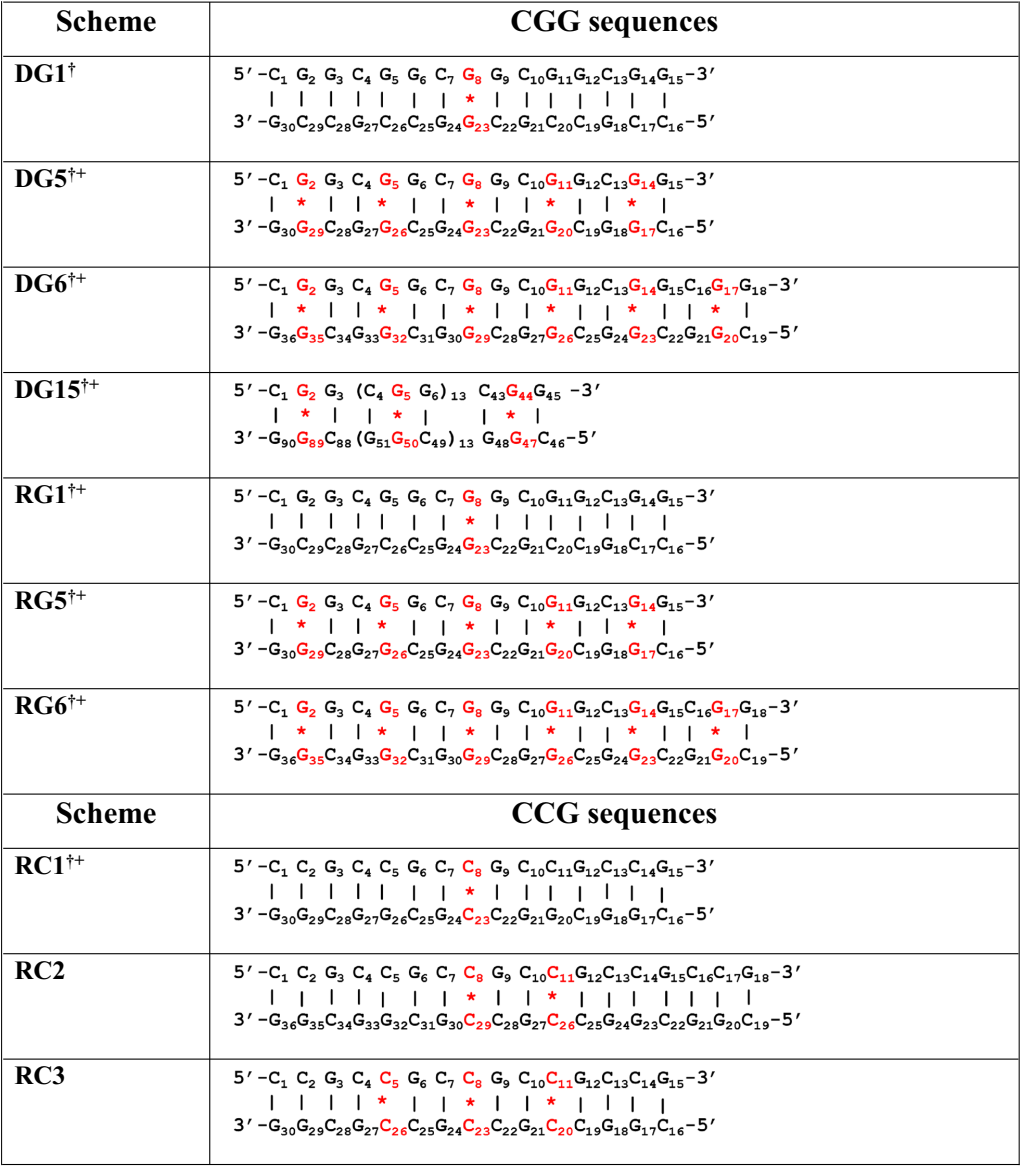

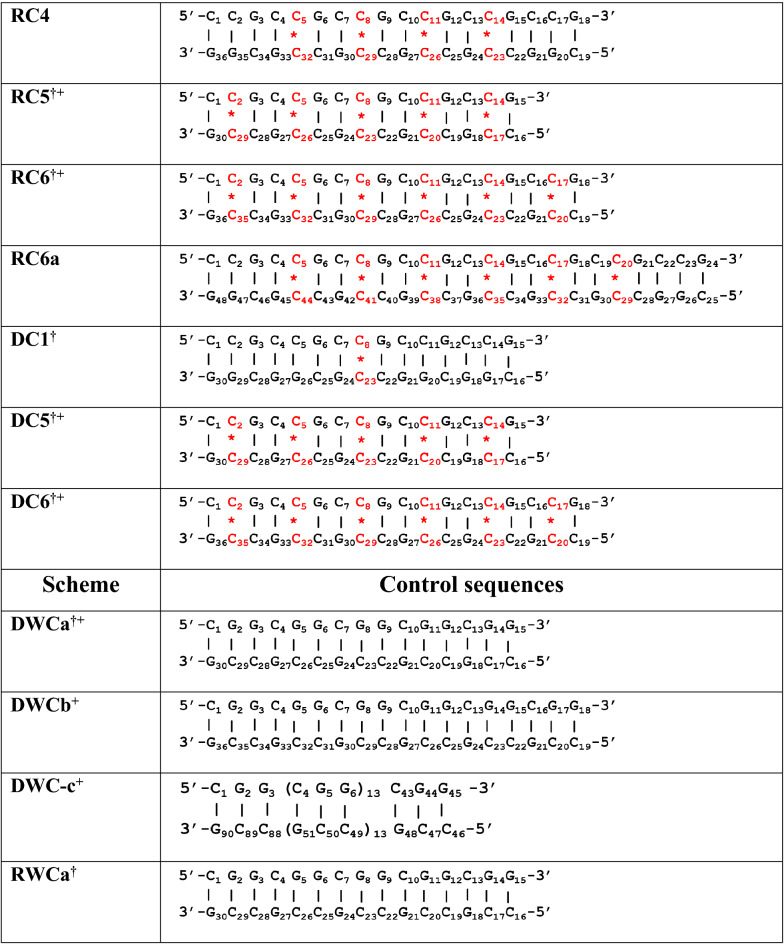
CGG and CCG repeats containing DNA and RNA duplexes considered for MD, EMSA and CD investigations.

The scheme name starting with R and D represents RNA and DNA, respectively and the numerals in schemes 1–7 represent number of mismatches.

The sequences indicated with ‘^**†**^’ are used for the CD experiments and the sequences indicated with ‘+’ are used in EMSA experiments. ‘*’ represents mismatched base pairs (colored red) and ‘|’ represents canonical base pairs.

### DNA and RNA CGG repeats favor a parallel quadruplex structure

**Figure 1:**
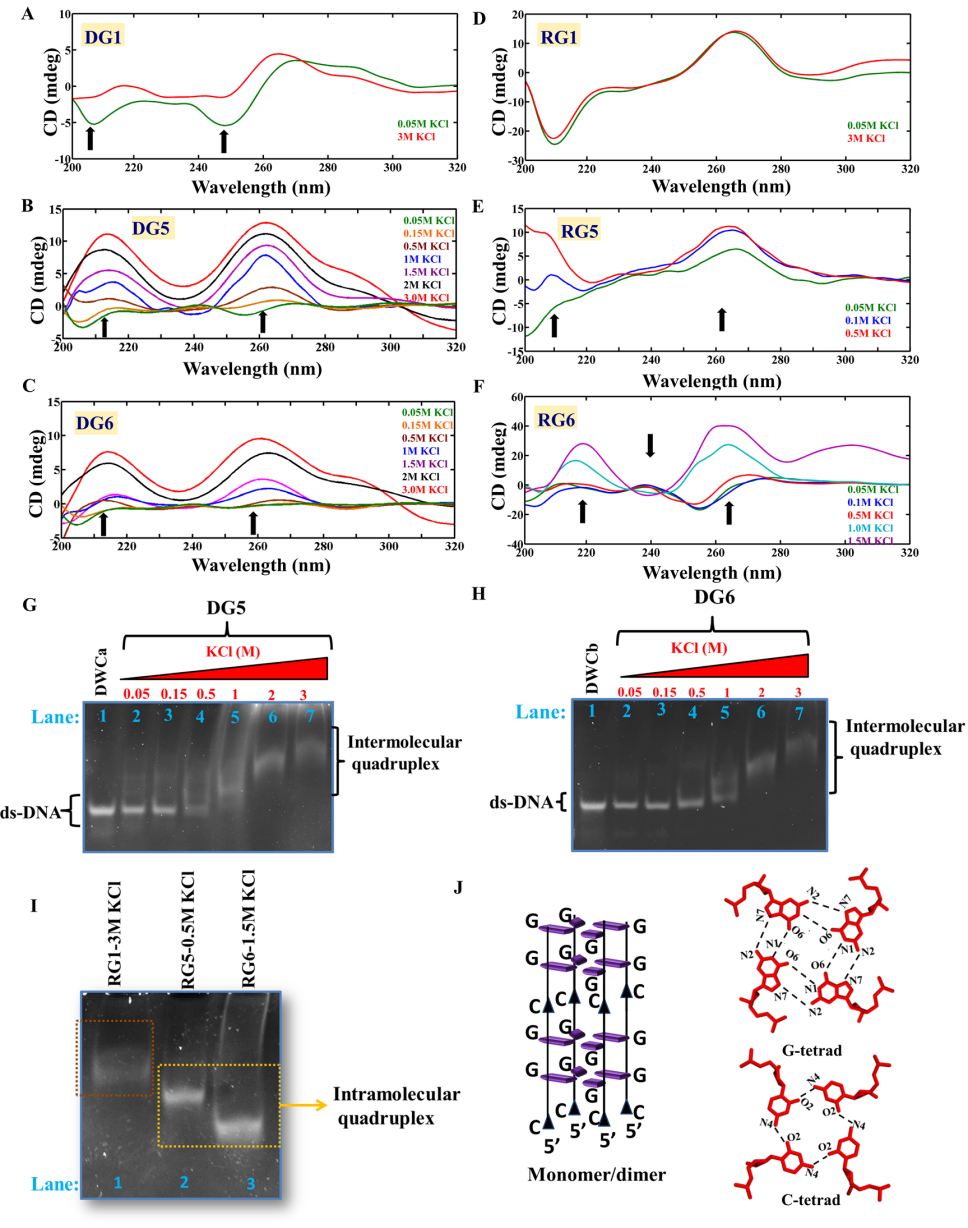
Circular dichroism spectra and EMSA corresponding to DNA and RNA CGG sequences. (**A**–**F**) CD spectra and (**G**–**I**) PAGE showing the preference for the following conformations by DG1, DG5, DG6, RG1, RG5, and RG6: (**A**) B-form duplex (0.05 M KCl)/intermediate conformation (3 M KCl) (DG1), (**B**,**C**,**G**,**H**) intermolecular quadruplex (DG5 and DG6), (**D**) A-form duplex (RG1) and (**E**,**F**,**I**) intramolecular quadruplex (RG5 and RG6). (**J**) Schematic diagram illustrating the arrangement of G- and C-quadrats (taken from PDB ID: 1EVO) in a parallel RNA and DNA CGG quadruplex. This figure was generated by using pymol 1.3 (www.pymol.com). The arrows indicate the increase or decrease in the ellipticity concomitant with the change in the secondary structure (see the text for more details). The figures (**A**–**F**) were plotted by using MATLAB 7.11.0 software (www.mathworks.com). The EMSA (**G**–**I**) samples were analysed by 14% native PAGE and stained with ethidium bromide (EtBr). The unprocessed gel images (**G**–**I**) are incorporated in Supplementary Fig. S13A–C.

CD experiments have been carried out for DNA and RNA sequences that are expected to form one (schemes DG1 & RG1 in Table 1), five (schemes DG5 & RG5) and six (schemes DG6 & RG6) G…G mismatches in a duplex. The CD spectra indicate that while the DG1 prefer B-form geometry (a positive peak at 275 nm and a negative peak at 255 nm^27^) (Fig. 1A), the DG5 (Fig. 1B) and DG6 (Fig. 1C) could not form a proper secondary structural conformation at a low KCl concentration. With an increasing KCl concentration (1–3 M), the CGG DNA given in DG5 and DG6 prefer a parallel quadruplex structure (Fig. 1B,C). The two positive peaks at ~ 215 nm and ~ 260 nm and a trough (instead of a negative peak) at ~ 240 nm at 1-3 M KCl concentration represent a parallel quadruplex formation in the case of DG5 and DG6^19,28,29^. Such a trough around 240 nm is an indication of higher order parallel quadruplex conformation as described in an earlier study^30^. Interestingly, an additional positive peak that is observed at 290 nm for DG6 (Fig. 1C) at higher concentrations of KCl (2 M and 3 M) may be because of the coexistence of a minor population of the hybrid quadruplex conformation^28,29,31^. The formation of quadruplex structure is further confirmed through the hypochromic thermal melting pattern, which is a characteristic feature of quadruplex structure^28,32^ (Supplementary Fig. S1). Interestingly, the DG1 sequence that forms B-form at a 0.05 M KCl concentration attains a conformation that is intermediate between B-form and quadruplex at 3 M KCl concentration. This can be seen from the negative peaks at 255 nm and 210 nm, which are absent in the 3 M KCl concentration (Fig. 1A). Not surprisingly, DG1 takes a B-form conformation at any concentration of NaCl in contrast to DG5 and DG6 as they are unable to form a defined secondary structure (Supplementary Fig. S2A–C), which is characteristic of a quadruplex structure ^19^.

The scheme for RG1 that is expected to form a duplex favor an A-form conformation with a positive peak at 265 nm and a negative peak at 210 nm (Fig. 1D, Supplementary Fig. S2D). However, RG5 exhibits an intermediate conformation between the A-form and quadruplex, as indicated by the absence of a negative peak at 210 nm (a signature peak of A-from). The ellipticity around 210 nm increases with an increasing KCl concentration (Fig. 1E). RG6 (isosequential to DG6) exhibits the characteristic features of a parallel quadruplex conformation, as indicated by the presence of a positive ellipticity at 220 nm and 260 nm and negative ellipticity at 240 nm with the increasing KCl concentration (Fig. 1F). However, RG5 and RG6 do not adopt any secondary structural conformation in the presence of NaCl (Supplementary Fig. S2E,F). A schematic representation of the possible quadruplex structure that can be formed by CGG repeats with G- and C-tetrads is shown in Fig. 1J. It is noteworthy that a recent study has reported that a water-mediated C-tetrad can easily be accommodated in a quadruplex^33^. To further confirm the CD results, we have carried out EMSA for both the DNA and RNA CGG repeats by varying the KCl concentrations. The DG5 (Fig. 1G) and DG6 (Fig. 1H) sequences exhibit lower mobility in the gel compared with the canonical duplexes (DWCa and DWCb) with increasing KCl concentrations. This clearly pinpoints the formation of intermolecular quadruplex conformation. EMSA further reveals that the B-form to quadruplex transition takes place between 0.5 and 1 M KCl, which is quite high compared with the normal physiological condition (~ 0.15 M KCl). However, in the case of a DNA sequence that has 15 CGG repeats (DG15), which is longer than DG5 & DG6, the transition from duplex to quadruplex conformation takes place at a ~ 0.15 M KCl concentration itself. This can be readily seen from the slower mobility of the band at a 0.15 M KCl concentration compared with the band corresponding to 0.05 M KCl (Supplementary Fig. S3). Thus, it is clear that the increase in the CGG repeat length may promote quadruplex formation at the physiological KCl concentration. Further, the slower migration of the DG5, DG6, and DG15 bands at the higher concentrations of KCl (1 M, 2 M, and 3 M) compared with the lower KCl concentrations (0.05 M, 0.15 M, and 0.5 M) indicates intermolecular quadruplex formation. As the *FMR1* gene undergoes expansion above 55 CGG repeats in premutated diseases, quadruplex conformation may be readily formed by the *FMR1* gene at physiological KCl concentration. Although RG6 favors quadruplex conformation (Fig. 1I) as the isosequential DNA, the nature of the quadruplex fold is different between the two. The faster migration of the RG5 and RG6 bands compared with the RG1 (15 mer duplex with a single G…G mismatch) band indicates the formation of an intramolecular quadruplex conformation in the former^34^. It is noteworthy that several X-ray and NMR studies have shown the ability of short oligonucleotide fragments (in the range of 15–20 nucleotide length) to form intramolecular quadruplex structures (PDB IDs: 2LK7, 2LYG, 2M6V, 2KOW and 1C35). In fact, the migration speed of RG5 is intermediate to that of RG1 (duplex) and RG6 (quadruplex), indicating that RG5 may take up an intermediate conformation. This result supports the CD data, which indicate that RG1, RG5, and RG6 take up A-form, intermediate, and quadruplex geometries, respectively (Fig. 1D–F). Thus, DG6 forms an intermolecular quadruplex conformation, while RG6 forms an intramolecular quadruplex conformation. One can envisage that such a quadruplex conformational preference by the r(CGG) and d(CGG) sequences with more number of CGG repeats may be due to the nonisomorphic nature of the G…G mismatch with the canonical G…C base pair. Thus, to investigate the structural distortions induced by the G…G mismatch in the RG6 and DG6 duplexes, we carried out MD simulations for these duplexes. Interestingly, irrespective of the two different AMBER force fields used in the simulations, both RG6 and DG6 retain the A- and B-form duplex conformations, respectively (Supplementary Fig. S4). The G…G mismatches are found to be stabilized by 2 hydrogen bonds (Supplementary Fig. S5). It is also possible that two such hairpin/duplex conformations can form a bimolecular antiparallel G-quadruplex structure with the formation of GGGG and GCGC tetrads, as found in a crystal structure (PDB ID: 1A6H). Thus, in the current investigation, EMSA and CD show the formation of a quadruplex conformation.

### Five and six C…C mismatches distort CCG RNA duplex

**Figure 2:**
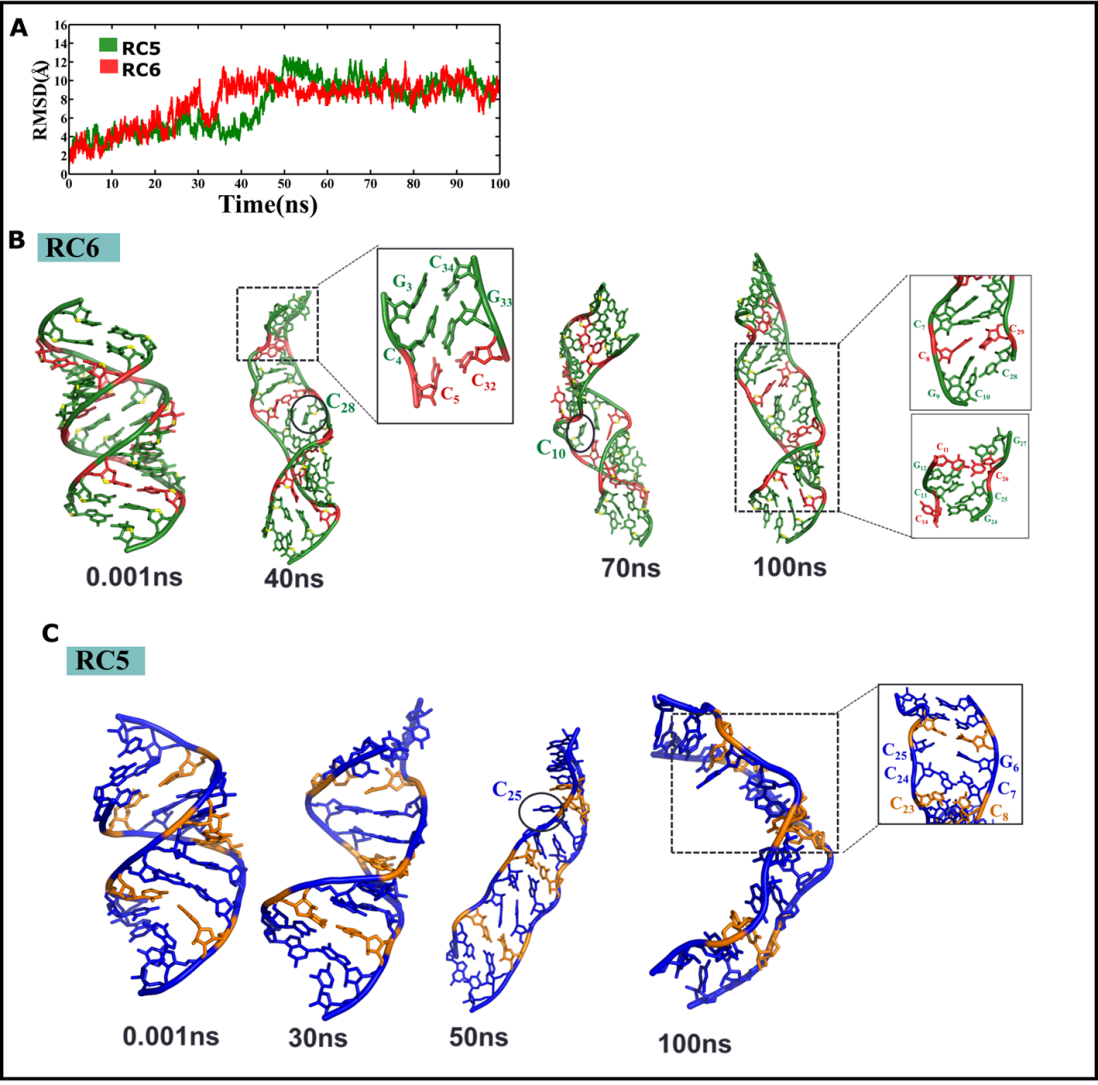
Schemes RC6 and RC5 that contain 6 & 5 C…C mismatches respectively, exhibit distortions in the double helix. (**A**) Time vs. RMSD profile showing significant conformational changes in RC6 (red color) and RC5 (green color) as indicated by a high RMSD value. This figure was plotted by using MATLAB 7.11.0 software (www.mathworks.com). (**B**,**C**) Snapshots illustrating the distortions in the double helix caused by the conformational rearrangement of C…C mismatches in RC6 (**B**) and RC5 (**C**). Note that the unpaired cytosines are shown in circles. This figure was generated by using pymol 1.3 (www.pymol.com).

Cumulative 0.9 microsecond MD simulations have been carried out for 7 CCG RNA duplexes that contain C…C mismatches in the range of 1 to 6. The duplex schemes used in the simulations are RC1, RC2, RC3, RC4, RC5, RC6 and RC6a (Table 1). To our surprise, during the 100 ns simulation, 6 C…C mismatches that periodically occur at every 3rd position of the RC6 duplex and are modelled to have a N3(C)…N4(C) hydrogen bond distort the A-form geometry. The RMSD value of 9 Å at the end of the simulation with respect to the initial model indicates that the final structure deviates more from the starting model (Fig. 2A). Such a high RMSD is the reflection of the structural distortions induced by the C…C mismatches in the duplex (Fig. 2B). Even during the earlier part of the simulation, the C…C mismatches are quite dynamic in such a way that many of the cytosines in the mismatch move either toward the major groove or toward the minor groove. This high flexibility, in fact, facilitates the establishment of the canonical C…G base pairing between one of the cytosines engaged in the C…C mismatches with the adjacent guanines (involved in canonical G…C hydrogen bond). This results in the alteration of the hydrogen bonding pattern in the CCG RNA duplex, leading to distortions in the helix. One such example is the distortion induced at the C_5_…C_32_ mismatch site around 7.3 ns. Due to the highly dynamic nature of C_5_…C_32_, C_5_ pairs with the adjacent G_33_ and forms the canonical C_5_…G_33_ base pair. As a result, C_4_, which is originally paired with G_33_, establishes the noncanonical hydrogen bond with the flanking C_34_. This eventually leaves C_32_ unpaired, causing distortions in the helix (Fig. 2B (40 ns), Zoomed view). Because of such movements, C_8_, C_10_, C_13_, C_25_, C_28_, and C_32_ are left unpaired at the end of the simulation (Fig. 2B (100 ns), Zoomed view). Similar distortions in the RC5 that contains 5 C…C mismatches can readily be seen with a high RMSD value of ~ 10 Å after 50 ns (Fig. 2A,C).

To confirm that the above mentioned helical distortions are mainly due to the dynamic nature of C…C mismatch and not due to the end fraying effect, 300 ns MD simulations have also been carried out for the RC6a scheme (Table 1). This duplex differs from RC6 just by an additional CCG trinucleotide that forms canonical base pairs on either end of the duplex. Although the helix is quite stable until 100 ns unlike RC6, the distortions in the helix are quite prominent after 200 ns (Supplementary Fig. S6). Thus, it is clear that 5 and 6 C…C mismatches induce distortions in the RNA double helix. Essentially, a similar distorting effect is seen for RC6 during the 500 ns MD simulations carried out using a different RNA AMBER force fields^35,36^ (Supplementary Figs. S7A,C, S8A–D).

### CCG RNA duplex can bear the brunt of 4 C…C mismatches

**Figure 3:**
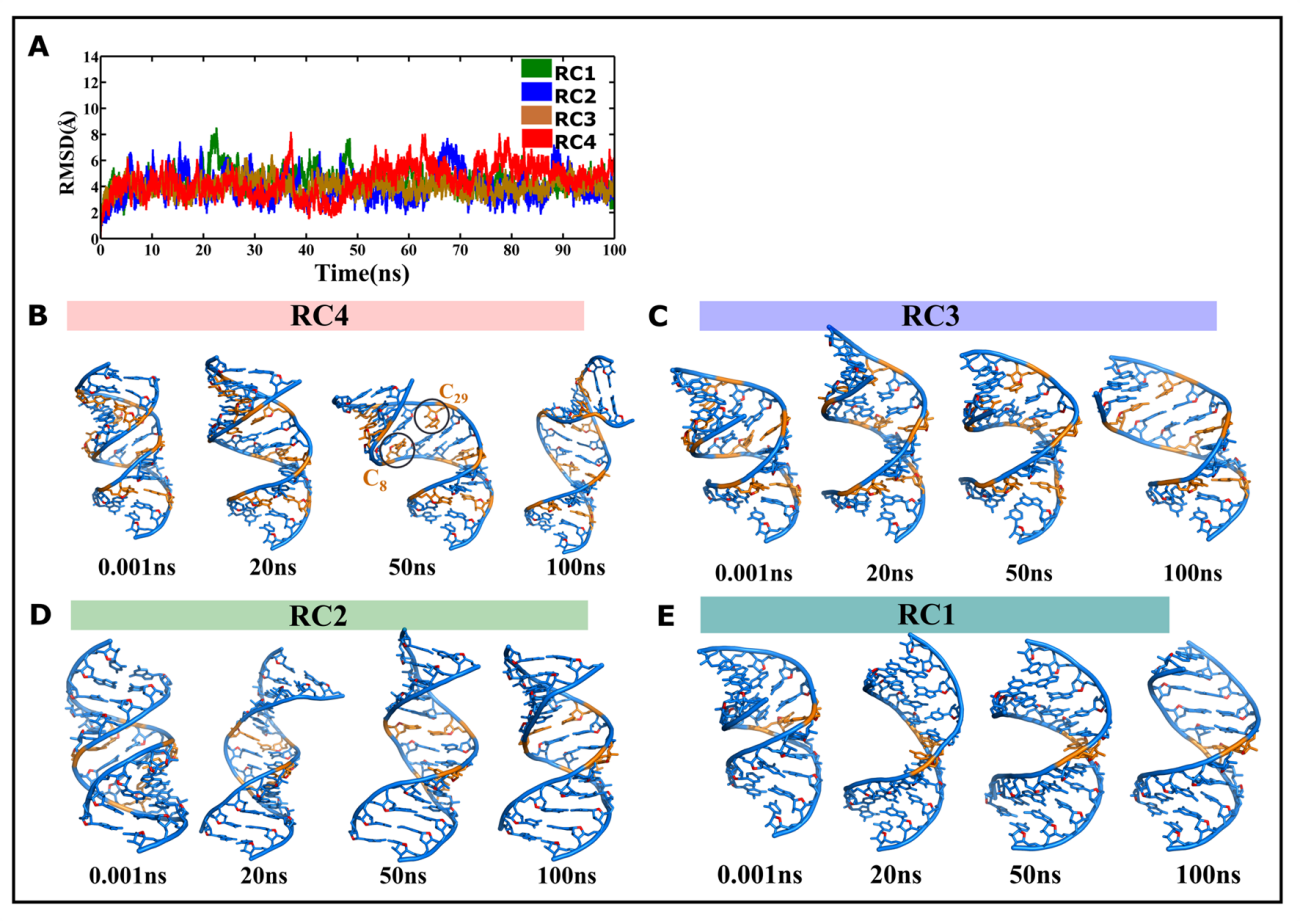
CCG RNA duplexes that contain 1 to 4 C…C mismatches are quite stable. (**A**) Time vs. RMSD profile showing less conformational changes in the RNA duplexes that contain one to four C…C mismatches. This figure was plotted by using MATLAB 7.11.0 software (www.mathworks.com). Cartoon representation of the snapshots corresponding to (B) RC4 (4 C…C mismatches), (**C**) RC3 (3 C…C mismatches), (**D**) RC2 (2 C…C mismatches) and (**E**) RC1 (1 C…C mismatch). Orange colored base pairs represent C…C mismatches (**B**–**E**). This figure was generated by using pymol 1.3 (www.pymol.com).

In addition, the 100 ns MD simulation have been carried out for the RC4 scheme (Table 1) that contains 4 C…C mismatches, wherein both the cytosines are base paired through a N3(C)…N4(C) hydrogen bond. The RMSD value of ~ 4 Å (calculated with respect to the starting model) observed during the simulation clearly indicates that the strand distortions caused by 4 C…C mismatches in the RNA duplex are quite insignificant (Fig. 3A) compared with 5 and 6 C…C mismatches (Fig. 2A).

Although the distortions in the C…C hydrogen bond are observed transiently due to the movement of cytosines toward the major or minor groove, as seen in C_8_…C_29_ around 50 ns, an A-form geometry is retained in RC4 (Fig. 3B). It is noteworthy that RC1, RC2, and RC3, which contain 1, 2, and 3 C…C mismatches, respectively, have also retained an A-form geometry (Fig. 3C–E).

### CCG repeat containing DNA duplex retains the B-form geometry irrespective of the number of C…C mismatches

**Figure 4:**
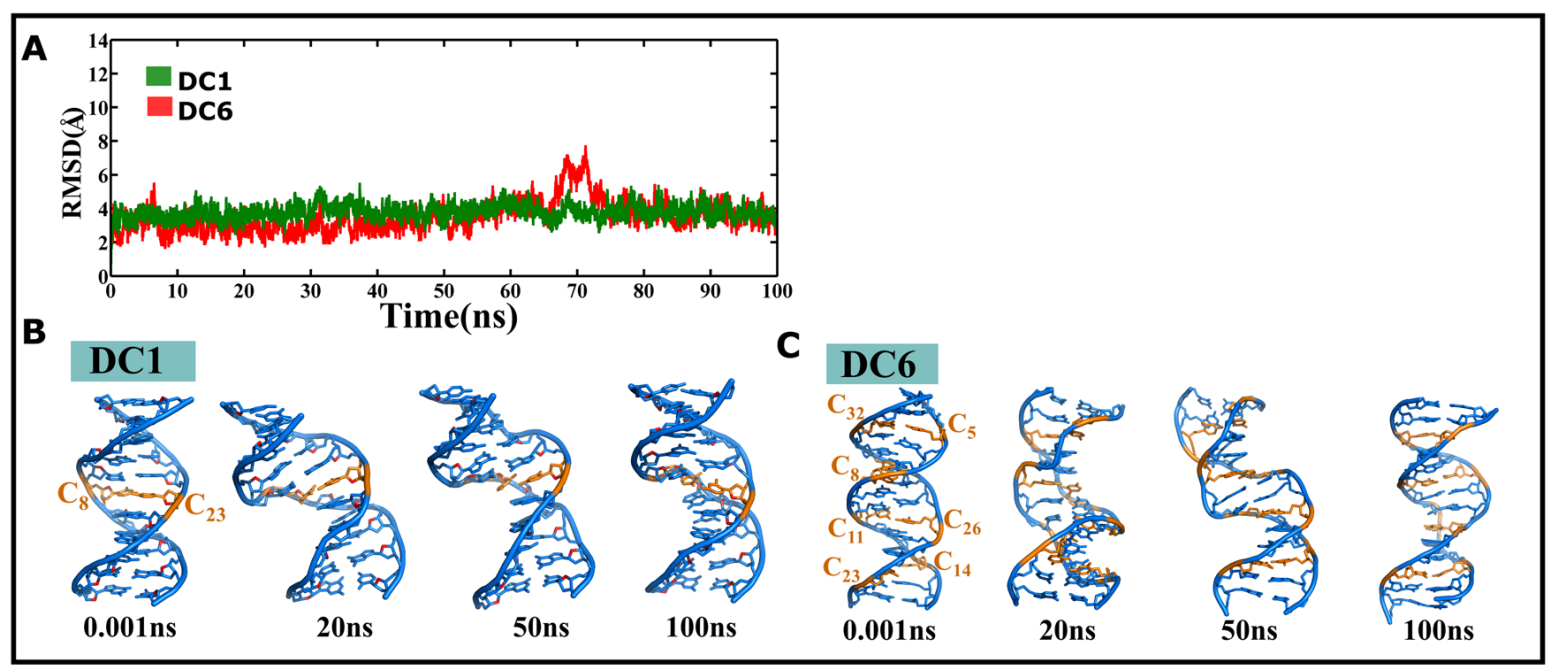
CCG DNA duplexes that contain 1 and 6 C…C mismatches are quite stable. (**A**) Time vs. RMSD profile corresponding to DNA duplexes that have one (green color) and six (red color) C…C mismatches. The MATLAB 7.11.0 software (www.mathworks.com) was used to plot the data. Note that the lower RMSD value of 4 Å indicates the stable nature of the duplexes. Snapshots corresponding to DNA duplexes that contain (**B**) one and (**C**) six C…C mismatches. This figure was generated by using pymol 1.3 (www.pymol.com).

The DNA duplexes that consist of 1 (DC1) and 6 (DC6) C…C mismatches, respectively, show stable B-form geometry over the 100 ns simulations. The RMSD value calculated with respect to the initial model stays ~ 4 Å during the entire simulation (Fig. 4A). This indicates that the B-form geometry is retained throughout the simulation (Fig. 4B,C). In addition, a 500 ns MD simulation have been carried out using a different DNA AMBER force fields^35,37^ also shows that DC6 (6 C…C mismatches) can be tolerated in the CCG DNA duplex (Supplementary Fig. S7B,D) in contrast to the isosequential RNA duplex (Fig. 2B), wherein the C…C mismatches above 4 distort the A-form geometry.

### Preponderance of duplex/hairpin conformation by d(CCG) and i-motif conformational intermediates by r(CCG)

**Figure 5:**
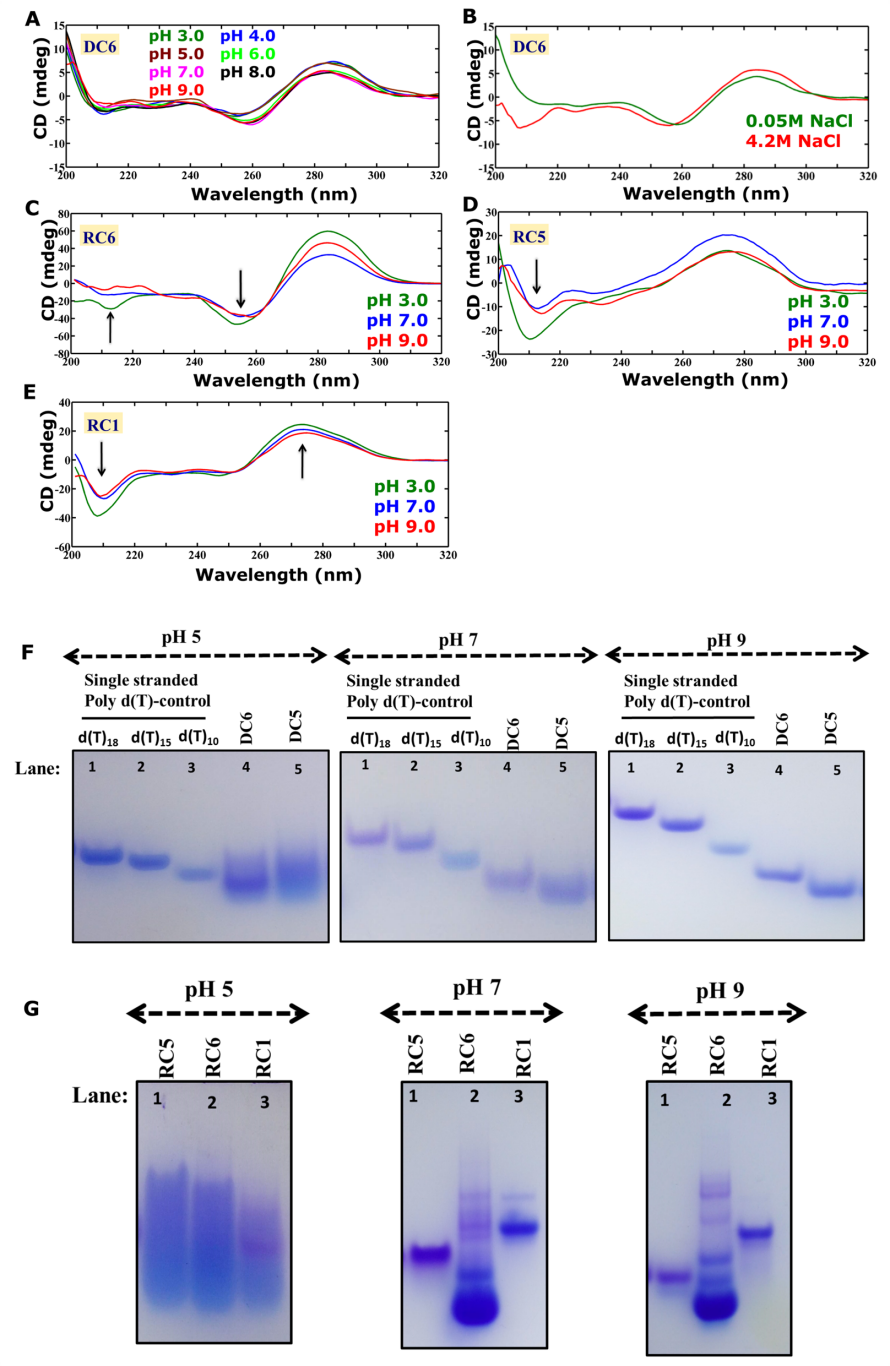
Circular dichroism spectra and EMSA corresponding to the DNA and RNA duplexes comprising of CCG repeats. (**A**) pH and (**B**) salt-dependent CD spectra showing the preference for duplex conformation by DC6 that contain 6 C…C mismatches. Preference for (**C**,**D**) i-motif like conformations by RC6 and RC5, and (**E**) A-form conformation by RC1. The figures (**A**–**E**) were plotted by using MATLAB 7.11.0 software (www.mathworks.com). Gel picture corresponding to (**F**) DC6 and DC5 (lanes: 4, 5) and (**G**) RC5 (lane: 1), RC6 (lane: 2), and RC1 (lane: 3) sequences at pH 5 (left), pH 7 (middle), and pH 9 (right). The EMSA (**F**,**G**) samples were analyzed by 10% native PAGE and stained with Stains All dye. The unprocessed gel images (**F**,**G**) are incorporated in Supplementary Fig. S13D,E.

In line with the MD simulations, the CD spectra corresponding to DC6 (6 C…C mismatches) also supports the formation of B-form geometry with a positive peak around ~ 285 nm and a negative peak around ~ 260 nm, irrespective of pH (pH 3, 4, 5, 6, 7, 8, and 9) (Fig. 5A). Additionally, the salt-dependent CD spectra do not show any B to Z transition under various concentrations of NaCl (0.05 M NaCl and 4.2 M NaCl) (Fig. 5B). These indicate the preference for B-form duplex by DC6. The RNA duplex containing 6 C…C mismatches (RC6) forms an i-motif/i-motif conformational intermediate structure with a positive and a negative signature peaks at ~ 285 nm and ~ 255 nm respectively, irrespective of the pH (3, 7, and 9) (Fig. 5C). However, the RC5 scheme that has 5 C…C mismatches shows a positive peak at ~ 275 nm and a negative peak at ~ 210 nm at different pH values (3, 7, and 9). In addition, a peak broadening is observed for RC5 between 230 and 250 nm for pH values in the range of pH 3.0 and pH 9.0 (Fig. 5D). Although the negative signature peak around 210 nm indicates the presence of the A-form conformation, peak broadening may reflect the presence of both i-motif and A-form conformations. Thus, RC5 may adopt an intermediate conformation that has the features of both A-form and i-motif geometries. However, the differences in CD spectra of RC5 and RC6 indicate that the RNA conformations may be different between the two cases. In contrast, CD spectra associated with the RC1 sequence (containing a single C…C mismatch) show a positive and negative peaks at ~ 275 nm and ~ 210 nm, respectively, representing the formation of A-form RNA duplex at different pH values (3, 7, and 9) (Fig. 5E). Thus, it is clear that the number of C…C mismatches is the deciding factor for the preference of the A-form duplex or i-motif/i-motif like conformation by r(CCG).

The CD spectra of DNA sequence with canonical base pairs (DWCa without C…C mismatches) that possesses the canonical base pairs indicate the presence of B-form conformation at various concentrations of KCl and NaCl. This can be seen by a positive peak at ~ 270 nm and a negative peak at ~ 250 nm (Supplementary Fig. S9A,B). Similarly, RNA with canonical base pairs (RWCa without C…C mismatches) forms an A-form in the presence of KCl and NaCl (Supplementary Fig. S9C,D). Thus, the CD results support the MD observations.

To further support our CD and MD results, we have carried out EMSA for both DNA (DC5 & DC6) and RNA (RC1, RC5, and RC6) sequences. The results reveal that DC6 (lane 4) and DC5 (lane 5) migrate faster than the single-stranded d(T)_18_ (lane 1), d(T)_15_ (lane 2), and d(T)_10_ (lane 3), which is indicative of the formation of an intramolecular-folded conformation at pH 5 (Fig. 5F (left)), 7 (Fig. 5F (middle)), and 9 (Fig. 5F (right)). As the CD spectra corresponding to DC6 (Fig. 5A) and DC5 (Supplementary Fig. S10) at pH 5, 7, and 9 represent the B-form geometry, the conformations observed at pH 5, 7, and 9 in EMSA may correspond to a hairpin. However, smeared bands at pH 5 may correspond to a minor population of other conformations, such as i-motif or i-motif-like conformations^38^. Notably, the extent of smear is more at pH 5 compared with the pH 7 and pH 9. The C…C mismatch in the hairpin may be stabilized by the N4…N3 hydrogen bond at pH 7 and pH 9, whereas it may be stabilized by 3 hydrogen bonds (N4…O2, N3^+^…N3, and O2…N4) at pH 5. Thus, more number of C…C mismatches can be tolerated in a B-form geometry without inducing much structural distortion in the helix, as seen in the MD simulations (Fig. 4C). In contrast, the EMSA bands correspond to RC5 and RC6 at pH 5 (Fig. 5G, (lanes 1 & 2)) exhibit smears to a greater extent compared with the isosequential DNA (Fig. 5F, left (lanes 4 & 5)). The multiple bands with different migrating capacities may reflect a variety of conformations, including inter- and intra-molecular i-motif-like/i-motif conformations. Surprisingly, even the RC1 sequence shows similar smear at pH 5 that is absent at pH 7 and pH 9 (Fig. 5G (middle & right), (lane 3)). This could be due to the fact that the C-rich strand of RC1 may tend to take up i-motif-like/i-motif conformations at pH 5. Nonetheless, the EMSA band corresponding to RC1 exhibits a slower migration with a well-defined isolated band compared with RC5 and RC6 at pH 7 (Fig. 5G, middle) and pH 9 (Fig. 5G, right). While RC5 takes up a single band at both pH 7 and pH 9, RC6 has multiple bands with different migrating capacities. Interestingly, the strong band corresponding to RC6 migrates faster than the RC5 band at pH 7 and pH 9. Further, RC5 migrates slower than RC6 at pH 7 and 9. These results clearly indicate that while RC1 is taking up an intermolecular (duplex) conformation, the other two (RC5 and RC6) may form i-motif conformational intermediates at pH 7 and pH 9 as also seen in the CD experiments (Fig. 5C–E).

## Discussion

CGG repeat expansion associated with the 5′ UTR region of the *FMR1* gene leads to neurodegenerative disorders such as FXS (also called FRAXA), FXTAS, fragile X-associated primary ovarian insufficiency (FXPOI), and fragile X-associated diminished ovarian insufficiency (FXDOR)^6,7,39,40^. The occurrence of CGG repeats in the range of 55–200 (premutated state) and above 200 (full mutated state) in the noncoding region of *FMR1* gene result in FXTAS/FXPOI/FXDOR and FRAXA, respectively^26,41^. Further, CGG expansion in the intronic regions of the *Zinc finger protein 713* (*ZFN713)* and *AF4/FMR2 family member 3* (*AFF3)* genes leads to fragile site 7A (FRA7A) and fragile site 2A (FRA2A), respectively^41^. In the FRAXA (the full mutation state), hypermethylation of CpG islands^42,43^ switches off the transcription and translation of the *FMR1* gene^41,42,44^, resulting in the loss of gene function.

In sharp contrast, in the FXTAS (the premutation state), the CpG islands in the *FMR1* gene are nonmethylated^42^, and complex mechanisms are shown to be involved in the pathogenesis of FXTAS. Neuropathology of FXTAS predominantly includes altered RNA processing, such as bidirectional (sense and antisense) transcription of the CGG repeat region^45^, aberrant RNA splicing^12^, formation of repeat RNA foci through the sequestration of RNA-binding proteins (RBPs)^46–49^, RAN translation to produce homopolypeptide aggregates corresponding to both sense and antisense transcripts^44,50^ and reduced^48,51^ translation of the gene product (loss of gene function). For instance, toxic mRNA gain-of-function takes place in FXTAS, as revealed by the elevated expression of *FMR1* mRNA^46^, along with the diminished expression of FMRP^11^. *FMR1* mRNA intranuclear inclusion is also found in brain tissue isolated from the post-mortem of FXTAS patients ^46^ and in mouse models^48^. In addition, the antisense *FMR1* CCG mRNA is shown to have elevated expression in FXTAS patients, which is similar to the sense CGG mRNA^45^. RAN translation of both sense and antisense transcripts of *FMR1* mRNA produce toxic poly P, poly R, poly A, and poly G aggregates as ubiquitin-positive inclusions^44,50^. Indeed, poly G and poly A aggregates produced due to RAN translation in the *FMR1* gene are found in *Drosophila*, cell cultures, and mouse models, as well as in FXTAS patient’s brain as ubiquitin-positive inclusions^52–55^.

Although one can envisage the role of unusual secondary structural preference by the expanded CGG/CCG repeat in *FMR1* sense and antisense strands and their mRNA transcripts in the above-mentioned biological alternations, there is no precise information about their secondary structural choice. In the current investigation, we are exploring the influence of the number of noncanonical base pairs on the secondary structural preference of CGG and CCG repeats to provide a structural basis of FXTAS by employing CD, MD, and EMSA techniques.

### CGG repeats favor quadruplex structure

CGG sequences are shown to take quadruplex^13^ and hairpin^14,15^ structures. For instance, one of the earlier studies on d(CGG)_n=2,4,8,16_ repeats shows the formation of a quadruplex structure at higher concentrations of K^+^ ions^17^. Both quadruplex^56^ and hairpin ^14^ structures are observed for RNA sequences with CGG repeats in the range of 17 and 20. Yet another biophysical study shows that RNA sequences that contain 19 to 45 CGG repeats can form stable hairpin structures in the presence of an AGG interrupt^57^. Until now, 6 crystal/solution structures of CGG repeat(s) have been deposited in the PDB. These include one DNA (PDB ID: 4HIV) and four (PDB ID: 2NCQ, 2NCR, 3R1C, and 3SJ2) RNA structures that have 1 to 3 CGG repeats and are shown to form a hairpin structure. In contrast, DNA sequences that have 2 CGG repeats connected by 3T’s (loop) are shown to form a bimolecular antiparallel G-quadruplex structure (PDB ID: 1A6H). Thus, the influence of the repeat number in deciding the secondary structure of the expanded CGG repeat still remains unclear.

Thus, the current study explores the conformational preference for DNA and RNA sequences given in the schemes DG1, DG5, DG6, RG1, RG5 and RG6 (which vary by the repeat length, Table 1) by employing CD, EMSA, and MD techniques. Both the DNA and RNA sequences favor B- and A-form duplex respectively, when the number of G…G mismatches is one. However, they tend to adopt a parallel quadruplex conformation when the CGG repeats are 5 and 6 (Fig. 1). A similar kind of parallel quadruplex structure formation is observed for the r(G_4_C_2_)_4_ sequence in an earlier study, which is indicated by the presence of positive peaks at ~ 265 nm and ~ 200 nm^58^. The inability to form any stable conformation at low concentrations of KCl and in the presence of NaCl (Fig. 1, Supplementary Fig. S2) is yet another confirmation for quadruplex formation, a trend reported for G-rich sequences^17^. Similarly, RNA also adopts a stable quadruplex conformation in the presence of KCl but not in the presence of NaCl (Fig. 1E,F and Supplementary Fig. S2E,F). The preference for quadruplex conformation by the CGG repeats in DNA and RNA sequences are further confirmed by EMSA (Fig. 1G–I). While DG5 and DG6 take up an intermolecular quadruplex structure (Fig. 1G,H), the isosequential RNA forms an intramolecular quadruplex structure (Fig. 1I) ^34^. Additionally, EMSA shows that a longer DNA sequence with 15 CGG repeats (DG15) forms a parallel quadruplex structure (in contrast to the control duplex, scheme DWC-c) as also confirmed by CD spectra (Supplementary Fig. S3). In support of the EMSA, the thermal melting profiles clearly indicate a hypochromic pattern (a signature of quadruplex) (Supplementary Fig. S11). Thus, it is clear that when the number of CGG repeats increases, the formation of quadruplex structure is favored. Thus, when the CGG repeat number increases in FXTAS, the quadruplex structure is favored.

Intriguingly, the MD simulations carried out for DG6 and RG6 indicate that irrespective of the 2 different AMBER force fields, the 6 G…G mismatches do not induce significant conformational changes in the duplex (Supplementary Figs. S4, S5). This is not surprising because the residual twist and radial difference, the measures of base pair nonisostericity^59–62^, between the G…G and G…C base pairs (Supplementary Fig. S12) are insignificant compared with that of A…A and G…C base pairs. Interestingly, an A…A mismatch flanked by G…C/C…G base pairs induces a B-Z junction in a DNA duplex^63–66^. It is also possible that two such hairpin/duplex conformations can form a bimolecular antiparallel quadruplex structure with the formation of GGGG and GCGC tetrads, as found in a crystal structure (PDB ID: 1A6H). Thus, the reluctance to take up a duplex conformation by CGG sequences with more number of CGG repeats perhaps due to the sequence effect rather than the nonisostericity of G…G base pairs with the flanking canonical base pairs. It is noteworthy that CD spectra corresponding to the canonical base pairs (DWCa & RWCa) show the formation of B-form and A-form geometry, respectively, for the DNA and RNA in the presence of KCl and NaCl (Supplementary Fig. S9). Thus, this evidence suggests that the formation of quadruplex structures occurs in the case of CGG repeat expansion both at the DNA and RNA levels.

### Differential influence of C…C mismatch on the secondary structural preference of CCG DNA and CCG RNA

CCG repeats can form a hairpin structure with a periodic C…C mismatch at every third position of the hairpin stem (viz., duplex)^20,67^ when CCG undergoes expansion. In fact, UV spectroscopic studies indicate that r(CCG)_17_ forms a hairpin structure, which is the least stable among all the CNG (wherein, N = A or G or U or C) repeats^14^. Similarly, an earlier study suggests that RNA sequences with 2 CCG repeats are prone to form a hairpin structure^21^. The CD spectra show that d(CCG)_12_ takes up a B-form conformation, but it changes to a Z-form duplex in the presence of aluminum ions^68^. Apart from the hairpin/duplex structure^20^, the CCG repeats can also favor i-motif structures at acidic pH^22^. The i-motif structure consists of two intercalating C…C base pair mismatches that are formed by 4 different strands at acidic pH^19,69,70^. This four-stranded i-motif structure has been reported for a d(T(CCG)_3_A) sequence that is stabilized by C…C^+^ and G…G mismatches^22^. In contrast, d(CCG)_2_^71,72^, d(GCC)_3_^73^, d(CCG)_15_^20^ are prone to adopt an ‘extrahelical’ structure in the minor groove side of the duplex, the so called e-motif structure. In fact, structural studies of short oligonucleotides that contain CCG repeats report the preference for duplex (PDB IDs: 1ZEX, 4E59, 2RPT, and 4J5V with 1 to 3 CCG repeats in DNA and RNA sequences), e-motif (PDB ID: 1NOQ with 2 CCG repeats in a DNA sequence), and i-motif (PDB ID: 4PZQ with 3 CCG repeats in a DNA sequence) structures. However, the above studies do not clearly pinpoint the structural basis for the conformational choice of CCG repeats. One can envisage that the number of C…C mismatches can play a role in deciding the secondary structure of CCG repeats. Thus, to investigate the tolerance for the maximum number of C…C mismatches in a DNA duplex and an RNA duplex, MD simulations carried out for duplexes with one to six C…C mismatches (Table 1). Because of the flexible nature of the single hydrogen bonded C…C mismatch and the availability of a wider space in the A-form RNA duplex^74^, some of the cytosines in RNA duplexes with 5 (RC5) and 6 (RC6&RC6a) C…C mismatches are left unpaired because of the movement of the cytosines toward the major groove or the minor groove (Fig. 2B,C, Supplementary Figs. S6B, S7C) and distort the helix significantly. The current study has also reported that one of the cytosines in the C…C mismatches is unaligned with respect to other base pairs of the helix by completely moving toward the major groove (Fig. 2C). In contrast, an A-form geometry is observed for the RNA duplexes that have C…C mismatches below 4 (Fig. 3B–E), as also confirmed by CD (Fig. 5E). In support of the results obtained from the current investigation, the crystal structure of an RNA duplex that has 2 CCG repeats with 2C…C mismatches is shown to favor an A-form duplex^21^.

Interestingly, the CD results reveal the preference for i-motif conformational intermediates for RC6 (Fig. 5C)^75^ and for RC5 (Fig. 5D). In contrast, RC1 shows an A-form geometry (Fig. 5E). The EMSA results also clearly indicate that while RC1 is taking up an intermolecular (duplex) conformation, the other two (RC5 & RC6) (Fig. 5G) are forming the i-motif conformational intermediates at pH 5, 7, and 9, as seen in the CD (Fig. 5C–E). It is noteworthy that earlier studies have reported even the formation of i-motif conformation at the neutral pH^76,77^ and in vivo conditions^78,79^.

In sharp contrast, the MD results show that CCG repeats with six C…C mismatches can readily be accommodated in a DNA duplex without significantly distorting the B-form geometry (Fig. 4). CD spectra corresponding to CCG DNA clearly pinpoint the preference for the B-form geometry at different pH (3, 4, 5, 6, 7, 8, and 9) and salt concentrations (0.05 M NaCl and 4.2 M NaCl) (Fig. 5A,B). In addition, the EMSA results also reveal that DC5 (15mer) & DC6 (18mer) form a hairpin conformation as it moves faster compared with both d(T)_15_ and d(T)_18_ at pH 5, 7, and 9 (Fig. 5F). Notably, a minor population of other conformations (as indicated by band intensity) is also observed for both DC5 and DC6 at low pH. Thus, a CCG DNA duplex can accommodate more number of C…C mismatches in contrast to the CCG RNA duplex at pH 5, 7, and 9 (Fig. 5C,G).

### The preference for quadruplex or i-motif intermediate conformations by CGG or CCG repeats explains the pathogenesis of FXTAS

The pathogenic mechanisms associated with FXTAS are as follow: loss of *FMR1* gene function^11^, *FMR1* RNA gain-of-function^80,81^, and *FMR1* RAN translation^52,82^. Here, we have proposed a possible molecular basis for these pathogenic mechanisms based on the results discussed above combined with the existing in vitro and in vivo data.

**Figure 6:**
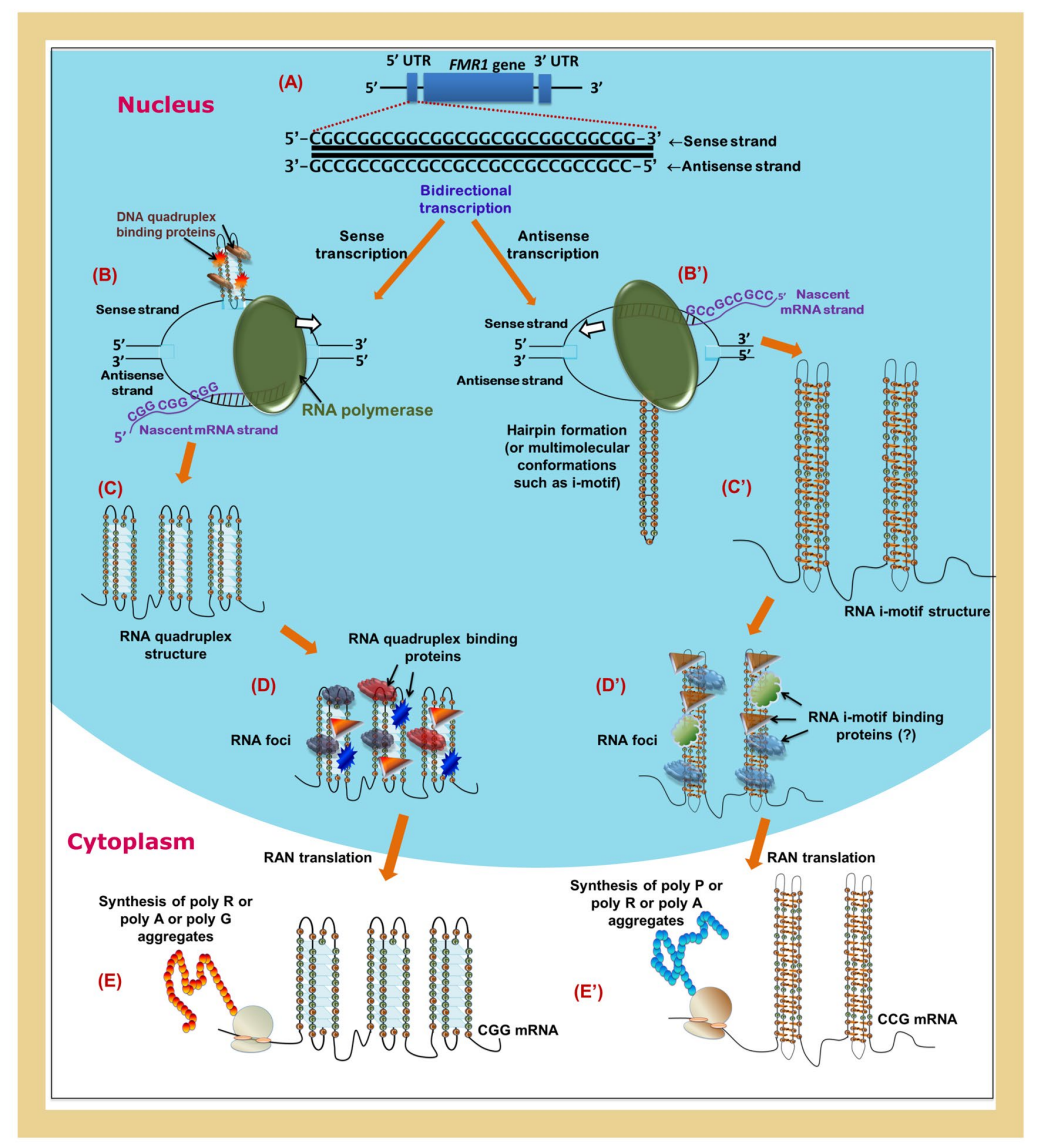
Proposed mechanism for the pathogenesis of FXTAS. (**A**) Expansion of CGG/CCG repeats in the 5′ UTR facilitates quadruplex (sense strand) and hairpin (antisense strand) formation. (**B**) Extended stability for the R-loop facilitates (**C**) quadruplex formation in *FMR1* CGG mRNA due to the stalling of RNA polymerase. (**D**) RNA quadruplex-binding proteins facilitate RNA foci formation and (**E**) promote RAN translation to synthesize poly R or poly A or poly G aggregates. (**B′**–**E′**) RNA misprocessing and RAN translation associated with CCG antisense *FMR1* mRNA. CCG repeats in RNA translate to either poly P or poly R or poly A aggregates. This figure was generated using Inkscape 0.91(https://inkscape.org/) software.

As per our CD and EMSA experiments (Fig. 1), it is clear that the CGG repeat in the *FMR1* gene (sense strand) forms a parallel quadruplex conformation. In line with this, earlier studies have shown that bimolecular quadruplex telomeric DNA-binding protein 42 (qTBP42) and unimolecular quadruplex telomeric DNA-binding protein 25 (uqTBP25) recognize and destabilize d(CGG) tetraplex^83^. Similarly, cationic porphyrin TMPyP4 is found to destabilize d(CGG) tetraplex^84^. Such a quadruplex formation in the *FMR1* gene (Fig. 6A) may stall the progression of RNA polymerase (Fig. 6B), providing an extended stability to the R-loop, which subsequently may facilitate frequent formation of quadruplex in CGG RNA (sense transcript). This subsequently may lead to the accumulation of abortive transcripts and result in the loss of gene function.

Further, we have shown here that the CGG RNA (sense transcript) has also been prone to form a quadruplex (Fig. 6C). The formation of a quadruplex by the CGG mRNA may form RNA foci (Fig. 6D) by sequestering the RNA-binding proteins and preclude their normal functions as also seen in GGGGCC repeat expansion^85,86^. Indeed, a recent in vivo experimental result shows that such RNA G-quadruplex formation is responsible for the neuronal dysfunction in FXTAS^87^. Such an RNA gain-of-function mediated by quadruplex formation may be the reason for the nuclear inclusions observed in the fly model^88^, animal models^89^, and FMR1 premutation patients^7,90^. In support of this, it has been shown in vivo that heterogeneous nuclear ribonucleoprotein (hnRNP) A2 or CArG-box binding factor A (CBF-A) (CGG quadruplex destabilizing proteins) significantly raises the efficacy of (CGG)_99_ mRNA translation in HEK293 cells, while the mutants of hnRNP A2 or CBF-A that lacks quadruplex-disrupting activity does not promote (CGG)_99_ mRNA translation^56^. Strikingly, hnRNP A2 is one among the protein found in the FXTAS inclusion^91^ along with the *FMR1* mRNA itself^46^. Interestingly, TMPyP4, which can unfold an extremely stable quadruplex^92^, is shown to cooperate with hnRNPs and increase the translational efficiency of fragile X premutation mRNA^93^. These clearly support in vivo quadruplex formation in the premutated CGG toxic RNA. FMRP, which is shown to bind to the parallel G-quadruplexes^94^, is also shown to recognize its own CGG mRNA^95^. Further, quadruplex formation may result in the aberrant translation of *FMR1* mRNA and may lead to RAN translation of polyG, polyA, and polyR, which are found in the ubiquitin-positive inclusion in the human brain of FXTAS patients^52,54,96^. A study has revealed that piperine, a known quadruplex-binding compound^97^, is shown to be effective in improving r(CGG)-associated splicing and RAN translation in a FXTAS cell model system^98^. Considering this, it is evident that quadruplex formation in *FMR1* transcript may be a cause for *FMR1*-premutation-associated diseases. Indeed, G-quadruplexes are generally found in a high density in the 5′ UTRs and play a regulatory role in post-transcriptional events^99^. In line with this, CGG repeats are found in the 5′ UTR of FRM1 gene, which upon expansion forms G-quadruplex structure. One can envisage that such a quadruplex formation may thus lead to aberrant post-transcriptional events and may be the cause of the RNA misprocessing events observed in FXTAS. Although some studies have shown that both RNA and DNA CGG repeats can form a hairpin structure, one cannot rule out the possibility that 2 such hairpins can come together and form an antiparallel quadruplex structure, as found in the atomic structure of DNA (PDB ID:1A6H) quadruplexes. Here, the quadruplex is stabilized through CGCG and GGGG quadrats instead of CCCC and GGGG quadrats, which are found in the parallel/hybrid quadruplex conformations (Fig. 1J).

The results presented in the current study also reveal the formation of the i-motif conformational intermediates structure by the antisense transcript. Similar to a quadruplex, such an i-motif or i-motif conformational intermediates secondary structure may also facilitate RNA foci formation and RAN translation (Fig. 6B′–E′). Thus, the formation of the quadruplex and i-motif or i-motif conformational intermediates structures may result in aberrant bidirectional translation of *FMR1* mRNA and antisense mRNA leading to RAN translation of polyG, polyA, and polyP, which are found in the ubiquitin-positive inclusion in the human brain of FXTAS patients^50,52,55^. Although d(CCG) favors the hairpin structure, the formation of bi/multimolecular i-motif structures cannot be ignored in the FMR1 premutated state, as reported earlier^22^. Thus, the pathogenic mechanisms presented here for FXTAS provide a convincing rationale for the molecular basis for FXTAS, as illustrated in Fig. 6. Although the model proposed here is based on the results obtained from the CD, MD and EMSA experiments (current study) as well as from the existing pathogenic mechanisms associated with FXTAS, there may be other unknow mechanisms associated with the FXTAS. Interestingly, the CCG repeat expansion occurring at the 5′end of the *FMR2* (*AFF2*) gene, which is associated with FRAXE syndrome, is shown to exhibit RAN translation in the premutated state in the *Drosophila* model^50,100^. Thus, the results presented here could be extended to FRAXE as well.

## Conclusions

The results presented here illustrate that CGG repeat expansion in the *FMR1* gene and the corresponding sense transcript form a quadruplex structure instead of a hairpin/duplex structure. Further, the corresponding antisense strand (CCG) has been shown to prefer a hairpin structure, and the antisense transcript is shown to prefer i-motif conformational intermediates structure due to its intolerance to more number of C…C mismatches in an A-form duplex. As quadruplex and i-motif structures are shown to be involved in transcriptional regulation, these secondary structural preferences reported here may have a role in altered the RNA processing and RAN translation seen in FXTAS. Combining the results presented here with the existing in vivo and in vitro data, we have presented here a convincing model that explains the neuropathology of FXTAS.

## Material and methods

### Molecular dynamics simulation

The initial models for the various DNA and RNA CCG duplexes (Table 1) were manually modeled using the Pymol suite (www.pymol.org, Schrödinger, LLC). The sequences were designed in such as a way that the mismatch containing CCG repeat should be flanked by equal number of CCG repeats on both the sides. This can be visualized from the sequences given Table 1. While a 15mer fulfils this requirement in the cases of odd number of C…C mismatches, an 18mer fulfils this requirement in the cases of even number of C…C mismatches. However, in the cases of RC4 (4 C…Cs) and RC6 (6 C…Cs) 18mer schemes, after ignoring the last 2 base pairs due to end-fraying effect^101^ they were eventually the same. Thus, to further capture the precise information about the influence of 4 and 6 C…C mismatches, an additional scheme (RC6a), an extension of RC6 scheme was designed. The scheme RC6a was designed in such a way to have an additional CCG repeat on both the sides of the helix to capture the pure effect of 6 C…C mismatches. All the sequences used in the MD simulations were designed in the perspective of capturing the influence of number of C…C mismatches. However, such a variety of sequences were not considered in the case of CCG DNA since there was no significant structural deformation observed between different schemes (DC1 and DC6 which were designed to have different number of C…C mismatches) during the MD simulation. The modeled duplexes were refined using constrained-restrained molecular geometry optimization using XPLOR-NIH^102^. Subsequently, the duplexes were solvated with a TIP3P water box and net-neutralized with Na^+^ counter ions. MD simulations were carried out under isobaric and isothermal conditions with SHAKE (tolerance = 0.0005 Å) on the hydrogen, a 2 fs integration time, and a cut-off distance of 10 Å for the Lennard–Jones interaction using the AMBER 12 suite^103^. The simulation was carried out at the neutral pH. The FF99SB force field (viz*.,* the default parm99.dat nucleic acid force field (without any correction) enabled through FF99SB option) was used for the simulation. The systems were initially equilibrated for 50 ps, following which the production runs were extended to 100 ns individually for the DNA and RNA duplexes, as given in Table 1. The MD simulations were carried out to a cumulative timescale of 1.1 μs. For the MD simulation of DNA (scheme DG6) and RNA (scheme RG6) CGG duplexes, the initial models were generated using 3D-NuS web server^104^. These duplexes were subsequently subjected to MD simulation following the protocol mentioned above. See Supplementary file for the details.

### Analyses of the trajectories

The Ptraj and cpptraj modules^105^ of AMBER 12 was used to post-process the MD simulation trajectories of the various DNA and RNA duplexes considered for the current investigation (Table 1). The root mean square deviation (RMSD) was calculated to acquire quantitative information about either the deviation or the proximity of the trajectories from the initial structure. MATLAB 7.11.0 (www.mathworks.com) software was used for plotting the graphs. Note that the two terminal residues at the 5′ and 3′ ends of the duplex were not considered for the analyses.

### Sample preparation

HPLC grade DNA and RNA oligonucleotides with CCG and CGG repeats (Schemes indicated by “^†^” in Table 1) were purchased from Sigma-Aldrich. The oligonucleotides (40 µM concentrations) were dissolved in KCl (0.05–3 M) or NaCl (0.05 M & 4.2 M) and with a 50 mM Tris–HCl/acetate buffer. The pH of the sample was in the range of 3–9 for the CCG oligonucleotides, whereas it was maintained at 7.4 for the CGG oligonucleotides. The DNA and RNA samples were initially heated to 95 °C for 5 min and subsequently cooled down to room temperature in a time period of 3 h. The secondary structure formation was verified by acquiring the CD spectrum. It is noteworthy that the CD spectra were collected immediately after the sample preparation because the quadruplex structures are prone to self-associate and form higher order structures^106^.

### CD spectroscopy

All CD spectra reported here were acquired in JASCO-1500 at 25 °C and processed using spectral manager software (ww.jascoinc.com). The data were collected in triplicate in the wavelength region of 200–320 nm and the baseline correction was done with respect to the appropriate buffer. All CD spectra corresponding to the triplicate average are reported here.

### Electrophoretic mobility shift assay

For the CGG samples, polyacrylamide gel electrophoresis (PAGE) was carried out using a 14% gel. The electrophoresis was carried out at 60 V for 3.5 h under cold conditions (4 °C). 1X TAE buffer was used to prepare the gel and the running buffer. Both the DNA and RNA samples were prepared with different concentrations of KCl (0.05 M to 3 M) and 50 mM Tris–HCl buffer (pH 7.4). Subsequently, a 25 µM concentration of the CGG RNA and DNA samples were mixed with 25% glycerol and loaded into the well. After running the electrophoresis, the PAGE gel (pretreated with ethidium bromide (EtBr)) was photographed under UV light using chemiDoc™ XRS from Biorad.

To run the electrophoresis for the DNA and RNA CCG samples, 10% polyacrylamide gel was prepared using 1× TAE buffer (pH 5, 7, and 9). Both the DNA and RNA samples were prepared in 50 mM NaCl and 50 mM Tris–HCl (pH 7 and 9) or Tris–acetate buffer (pH 5). As before, a 25 µM concentration of the CCG RNA and DNA samples was mixed with 25% glycerol and then loaded into the well; 1× TAE buffer (pH 5, 7, and 9) was used as the running buffer. Stains All (sigma) dye was used to stain the gel and photographed under a normal white light digital camera.

## Supplementary Information

Supplementary Information.
