# Supplementary material for: Secondary structural choice of DNA and RNA associated with CGG/CCG trinucleotide repeat expansion rationalizes the RNA misprocessing in FXTAS
Source: Sci Rep. 2021 Apr 14;11:8163. doi: 10.1038/s41598-021-87097-y (PMC8046799; doi:10.1038/s41598-021-87097-y)
Supplement: Supplementary file 1 — Supplementary Information. [file 41598_2021_87097_MOESM1_ESM.docx]

Supplementary information

**Secondary structural choice of DNA and RNA associated with CGG/CCG trinucleotide repeat expansion rationalizes the RNA misprocessing in FXTAS**

Yogeeshwar Ajjugal^#^, Narendar Kolimi^#^ and Thenmalarchelvi Rathinavelan*

Department of Biotechnology, Indian Institute of Technology Hyderabad,

Kandi, Telangana State-502285, India

^#^Equal contribution, *For correspondence: tr@iith.ac.in

*Running title*

Number of G…G/C…C mismatches dictates the secondary structure of (CGG)_n_ /(CCG)_n_

**Keywords: CGG/CCG repeat overexpansion, *FMR1* gene, *FMR2* gene, FXTAS, fragile X syndrome, circular dichroism, electrophoretic mobility shift assay, i-motif conformational intermediates, quadruplex, molecular dynamics simulation, RNA misprocessing, RAN translation, R-loop**

**MD simulations of DG6 and RG6 using a different AMBER force fields**

To compare the results obtained from the FF99SB force field with the OL15 (β_OL1_, εζ_OL1_, and χ_OL4_ corrections are added to ff99bsc0 for DNA) (1, 2) and OL3 (χ_OL3_ correction is added to ff99bsc0 for RNA) (1, 3) AMBER nucleic acids force fields, we carried out a 500ns production run simulation. Prior to this, a 1ns equilibrium simulation was carried out for the DG6, RG6, DC6, and RC6 sequences using the pmemd.cuda module of the AMBER 16 suite (4). The DG6, RG6, DC6, and RC6 duplexes were modeled using the 3D-NuS web server (5). Except for the force field, other simulation conditions are same as given in the Methods section of the main text.

**Results**

*G…G mismatch maintains the helicity of both CGG DNA and RNA*

To explore the structural changes induced by 6 G…G mismatches, we have carried out MD simulations for the DG6 and RG6 duplexes. Based on the crystal structure of RNA CGG repeat (PDB ID: 3R1C), *syn* and *anti* starting *glycosyl* conformations have been assigned for the G…G mismatches. First, we have proceeded with 100ns production run for DG6 with the FF99SB force field. Prior to this, we have carried out 70ps equilibrium run. Beyond 60ns, one of the mismatched guanine in G_23_…G_14_ undergoes base extrusion and persists until 100ns (Fig S4A). However, the helicity is retained in the B-form geometry. Similarly, B-form geometry is also retained throughout the 500ns simulation for DG6, even with the OL15 force field (Fig S4B).

Both the FF99SB and OL3 force fields retained the A-form geometry for RG6 (Fig S4C-D). Hydrogen bond analyses carried out for G…G mismatches indicate that the (G*_syn_*)N7…N2(G*_anti_*) and (G*_syn_*)O6…N1(G*_anti_*) hydrogen bonds are retained and are populated in the range of 2.5Å and 3.5Å distance for both DG6 and RG6 (Fig S5A-D). Exceptionally, a G23…G14 mismatch in the DG6 sequence has some minor populations in the range of 6.5Å and 8.5Å distance in the simulation carried out using the FF99SB force field because of the base extrusion event (Fig S5-A, right bottom). In any case, the helical geometry is retained for both DNA and RNA duplexes, irrespective of the force fields. Strikingly, the residual twist and radial difference, the measures of base pair nonisomorphism (6-8), are ~8.1° and ~0.7Å (Fig S12 left), respectively, between the G...G and G…C base pairs. This value is less significant compared with that of the A…A mismatch and G…C base pair (9, 10). Although MD simulations of the DG6 and RG6 duplexes with different force fields indicate stable hydrogen bonds between the G…G pair, CD and EMSA experiments show the formation of a quadruplex (see the main text). This indicates that such a preference for a quadruplex for both the DG6 and RG6 repeats are mainly because of the sequence effect.

*C…C mismatch destabilizes r(CCG)_6_ in contrast to d(CCG)_6_*

We further investigated the effect of the OL3 force field on the conformations of RC6. Although RMSD stays ~4Å, it increases to 6Å between 335 and 340ns due to a transient structural distortion (Fig S7A&C). Apart from such transient structural changes, no major destabilization of the RNA helical structure is observed, as seen in the case of FF99SB (Fig 2B). Instead, due to the highly dynamic nature of the C…C mismatches, there are significant fluctuations in their hydrogen bonds. Notably, the initial N3…N4 hydrogen bonds of the C…C mismatches are predominantly lost during the 500ns simulations (Fig S8A-D). However, a minor population of N4…O2 and O2…N4 hydrogen bonds is also found. Beyond 350ns, a total loss of at least three out of four central C…C mismatch hydrogen bonds are simultaneously observed in the helix. Such a loss of hydrogen bonds at every third position of the CCG RNA helix may reduce the stability of the helix compared with the cononical RNA duplex. This can be clearly seen in the hydrogen bond distance histogram, which occupies more populations in the range of 7–9Å compared with 2.5–3.5Å (Fig S8A-D). Among the 4 central C…C mismatches of RC6, the C_26_….C_11_ mismatch is very dynamic in nature, which results in the loss of hydrogen bonds. This reflects in the hydrogen bond distance histogram, wherein more populations are seen in the donor...acceptor distance in the range of 7–9Å (Fig S8C). Thus, it is clear that C…C mismatches in RC6 are dynamic in nature and move toward the minor groove or the major groove. This is despite the fact that the extent of a nonisomorphism of C…C mismatch with respect to the flanking canonical G…C base pair (residual twist=4.4° and radial difference=0.5Å (Fig S12, right)) is nearly similar to that of the U…U mismatch (residual twist=4° and radial difference=1.1Å), which easily gets accommodated in a CUG duplex (11). The availability of a wider space in the A-form geometry due to a large X-displacement may be the reason for the single hydrogen bonded C…C mismatch taking up a variety of conformations in contrast to the U…U mismatch, which is stabilized by 2 hydrogen bonds. Such a wide space is not feasible in the B-form geometry because of the negligible X-displacement, due to which the C…C mismatch hydrogen bonds are retained in DC6 (Fig S8E-H) during the MD simulations carried out using the OL15 force field. This is reflected in the RMSD that stays at ~2.8Å (retain the original) (Fig S7B). However, the local helical distortions are transiently observed during the simulations (Fig S7D). Eventually, similar results are observed in the MD simulations carried out using the FF99SB force field, wherein the C…C mismatch hydrogen bonds stabilize the helicity of DC6 (Fig 4C). Although, in RC6 the nature of the distortion is different between two different force fields (*viz.* the loss of hydrogen bond in OL3 versus the destabilization of helicity in FF99SB), it is clear that C…C mismatches destabilize RC6. Further, the CD and EMSA results confirm the MD observations (see the main text).

| 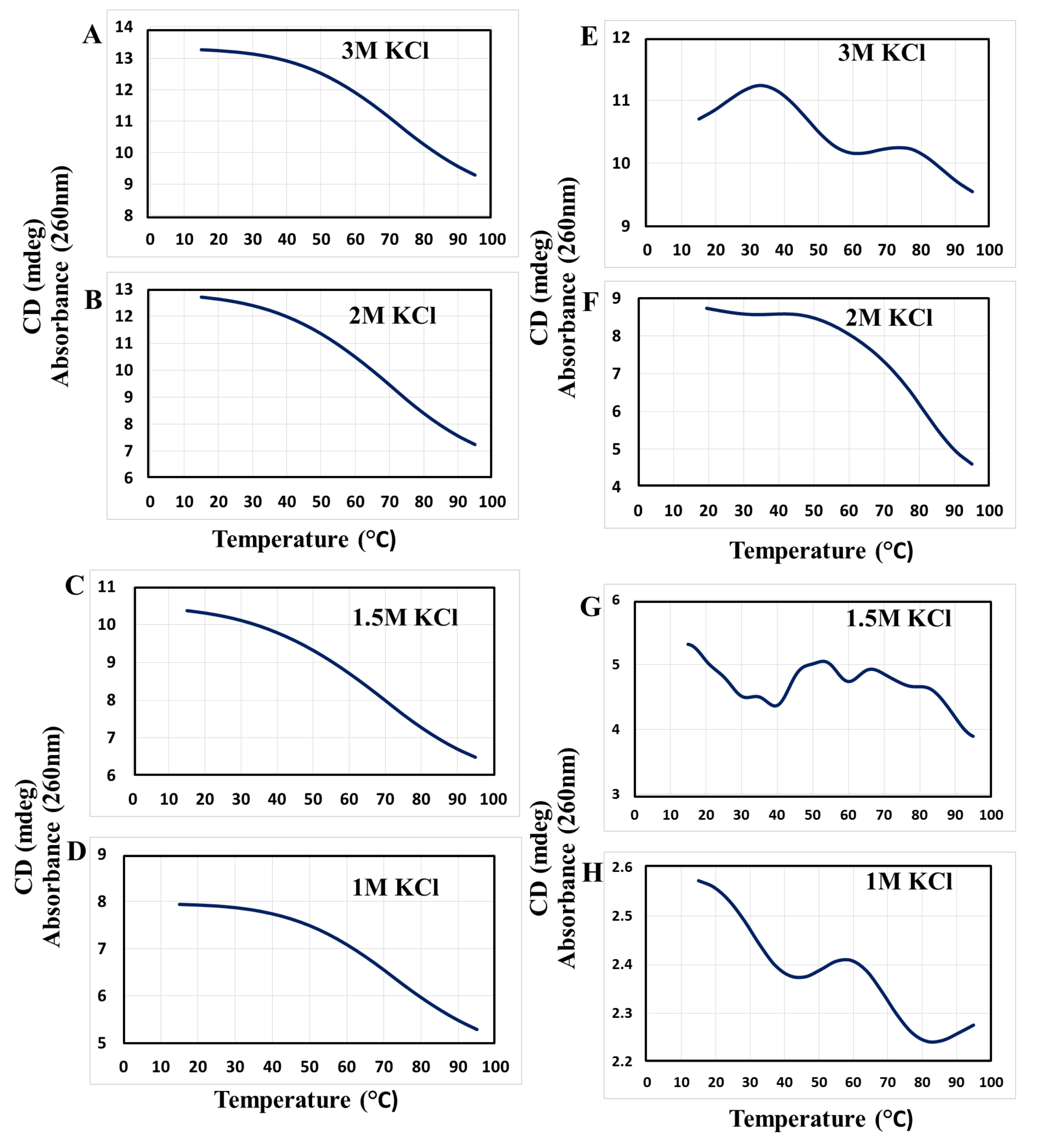 |
| --- |
| **Fig S1. Thermal melt corresponding to DG5 and DG6 at high concentrations of KCl.**  The thermal melting profiles collected at 1-3M KCl concentrations for DG5 (A-D) (15mer) and DG6 (E-H) (18mer) which confirm the formation of quadruplex. Note that the difference in the thermal melt profiles of the DG5(15mer) and DG6(18mer) may be attributed to the different quadruplex folds. |

| 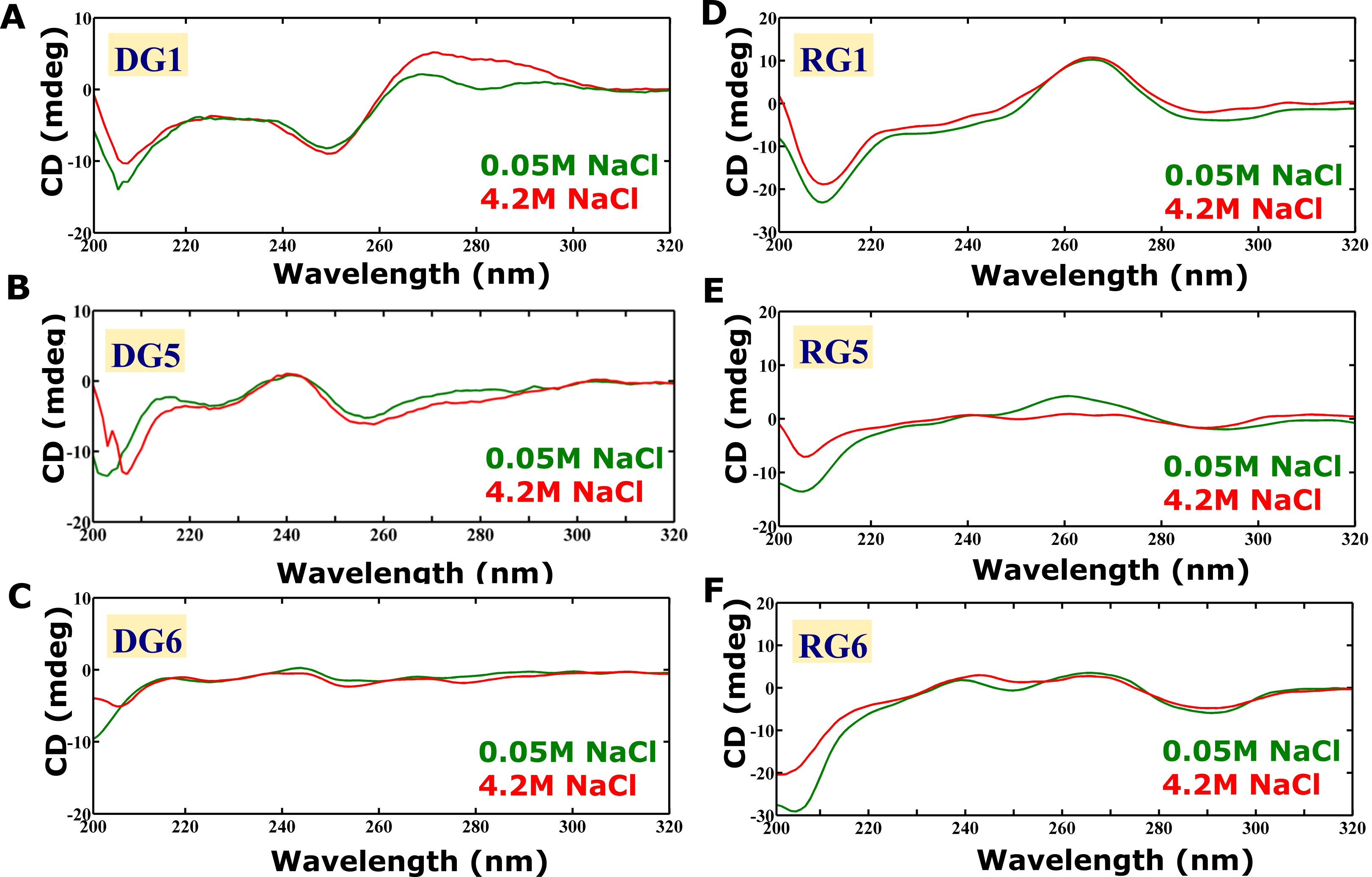 |
| --- |
| **Fig S2. CD spectra corresponding to DNA and RNA CGG oligonucleotides in the presence of low and high concentrations of NaCl.** (A) DG1 favor a B-form duplex structure with one G...G mismatch, whereas (B) DG5 and (C) DG6 do not form any proper conformation at the low and high concentrations of NaCl. (D) RG1 form an A-form duplex with one G...G mismatch, whereas (E) RG5 and (F) RG6 do not form any proper conformation at the low and high concentrations of NaCl. |

| 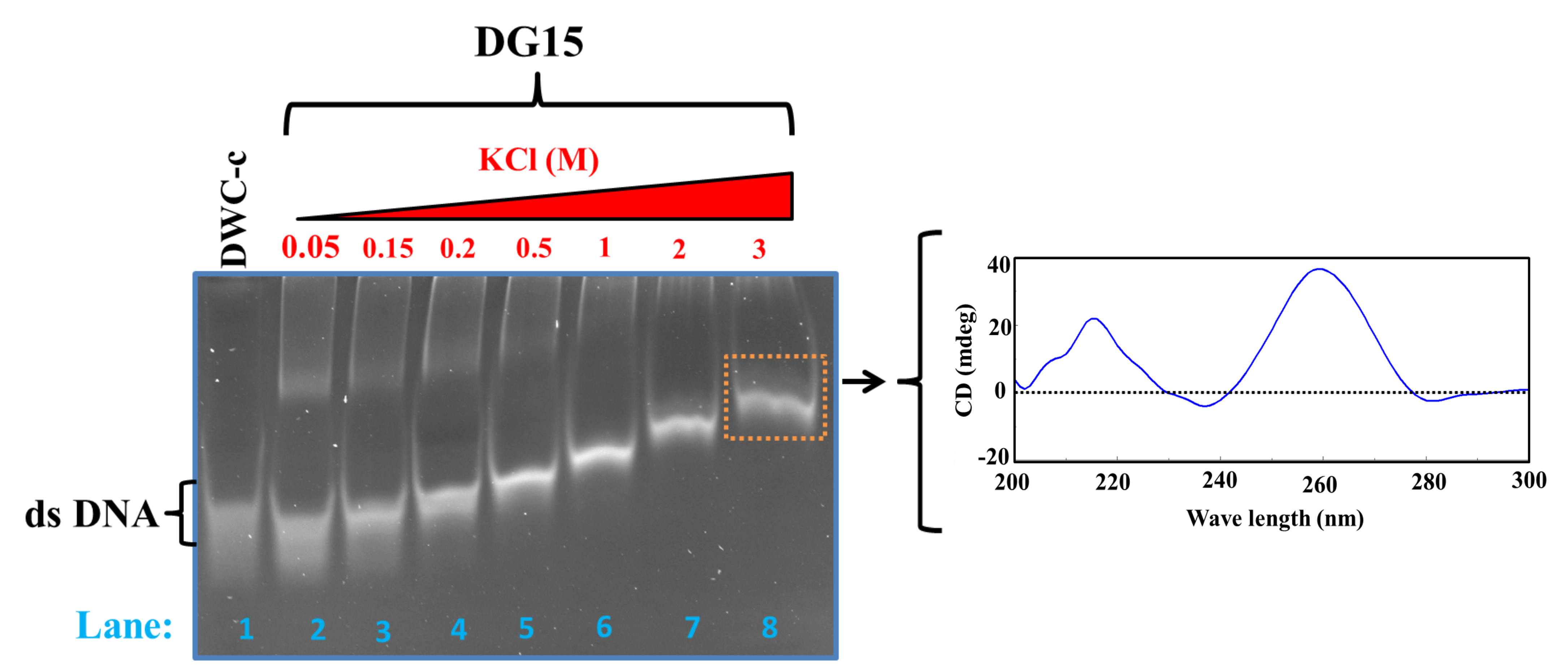 |
| --- |
| **Fig S3. EMSA for DG15 sequence with different KCl concentrations.** (Left) The transition from duplex to quadruplex is observed at physiological KCl concentration (lanes 2 and 3). The complete transition to a quadruplex conformation is observed at the higher KCl concentrations. (Right) CD spectra confirming the quadruplex formation. |

| 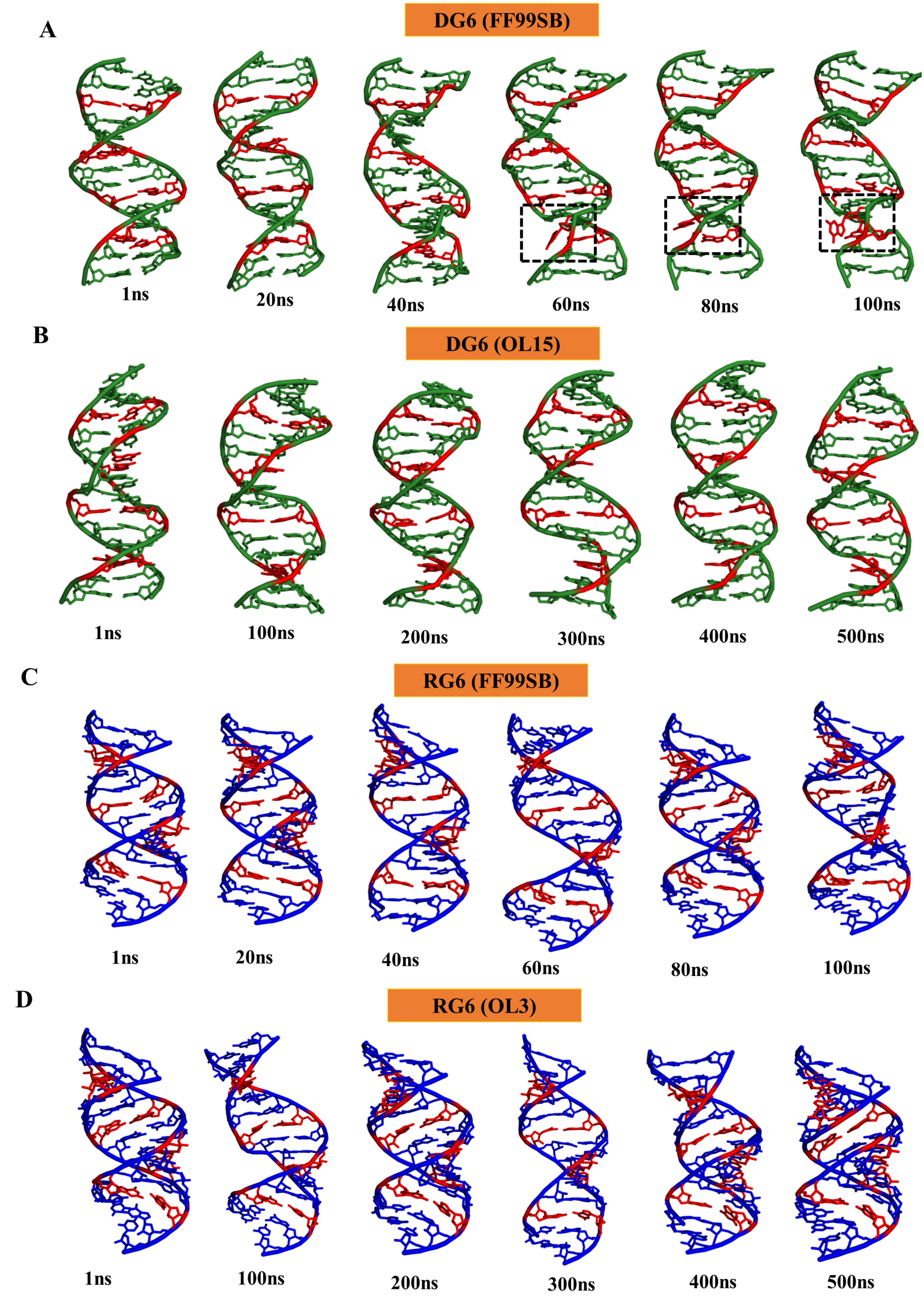 |
| --- |
| **Fig S4.** **Cartoon representation of (CGG)_6_ DNA and RNA duplexes showing the retention of helicity.** Snapshots corresponding to DG6 (6 G…G mismatches) (A-B) and RG6 (6 G…G mismatches) (C-D) obtained from the trajectories of MD simulations carried out using FF99SB (A, C), OL15 (B) and OL3 (D) force fields. Note that the G…G mismatches are colored red (A-D). |

| 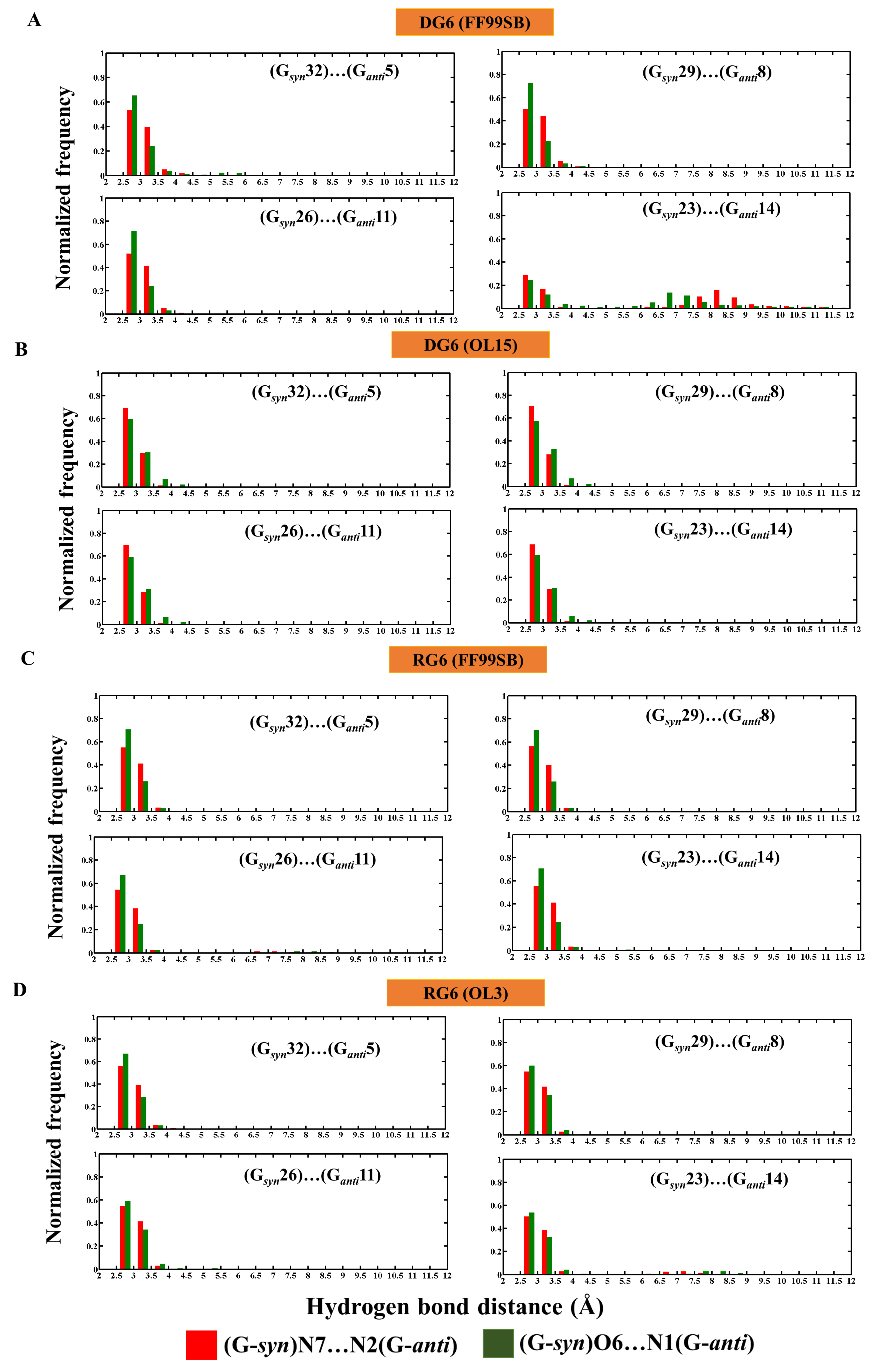 |
| --- |
| **Fig S5. Hydrogen bond distance distribution corresponding to the DNA and RNA (CGG)_6_ duplexes.** Hydrogen bond distance analysis corresponding to the central 4 G...G mismatch in DG6 (A-B) & RG6 (C-D) sequences, respectively. Irrespective of the force fields, the G…G mismatch hydrogen bond in both DG6 (6 G…G mismatches) & RG6 (6 G…G mismatches) are highly populated in the range of 2.5–3.5Å. Note that a terminal CGG triplet on either side of the duplex is excluded for the analysis because of the end-fraying effect. |

| 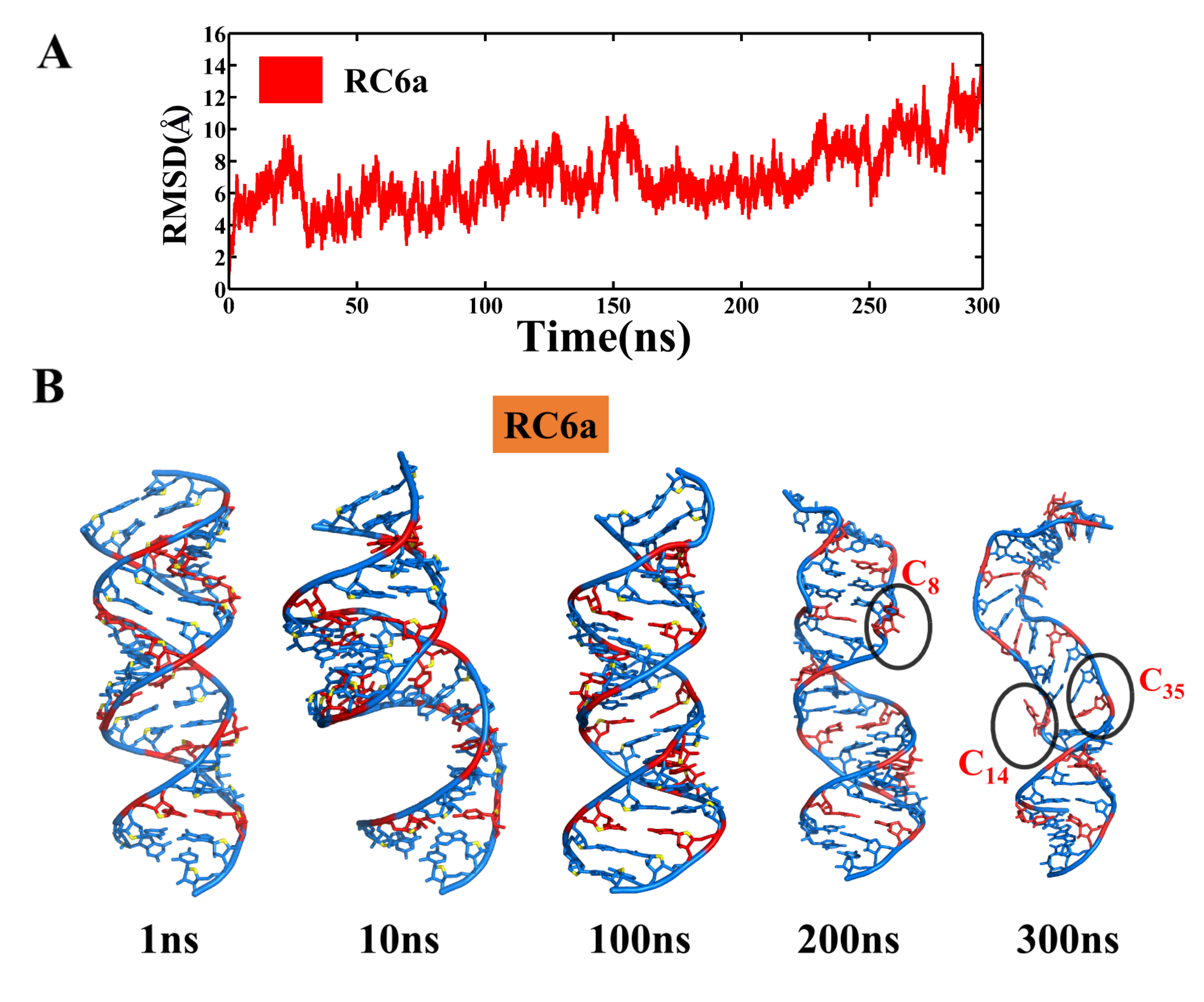 |
| --- |
| **Fig S6. RC6a that contains 6 C...C mismatches show distortions in the helix.** A) Time vs. RMSD profile showing significant conformational changes in RC6a, as seen by a high RMSD value. B) Snapshots showing the distortions in the helix because of the C...C mismatches in RC6a. Note that unpaired cytosines are shown in circles. |

| 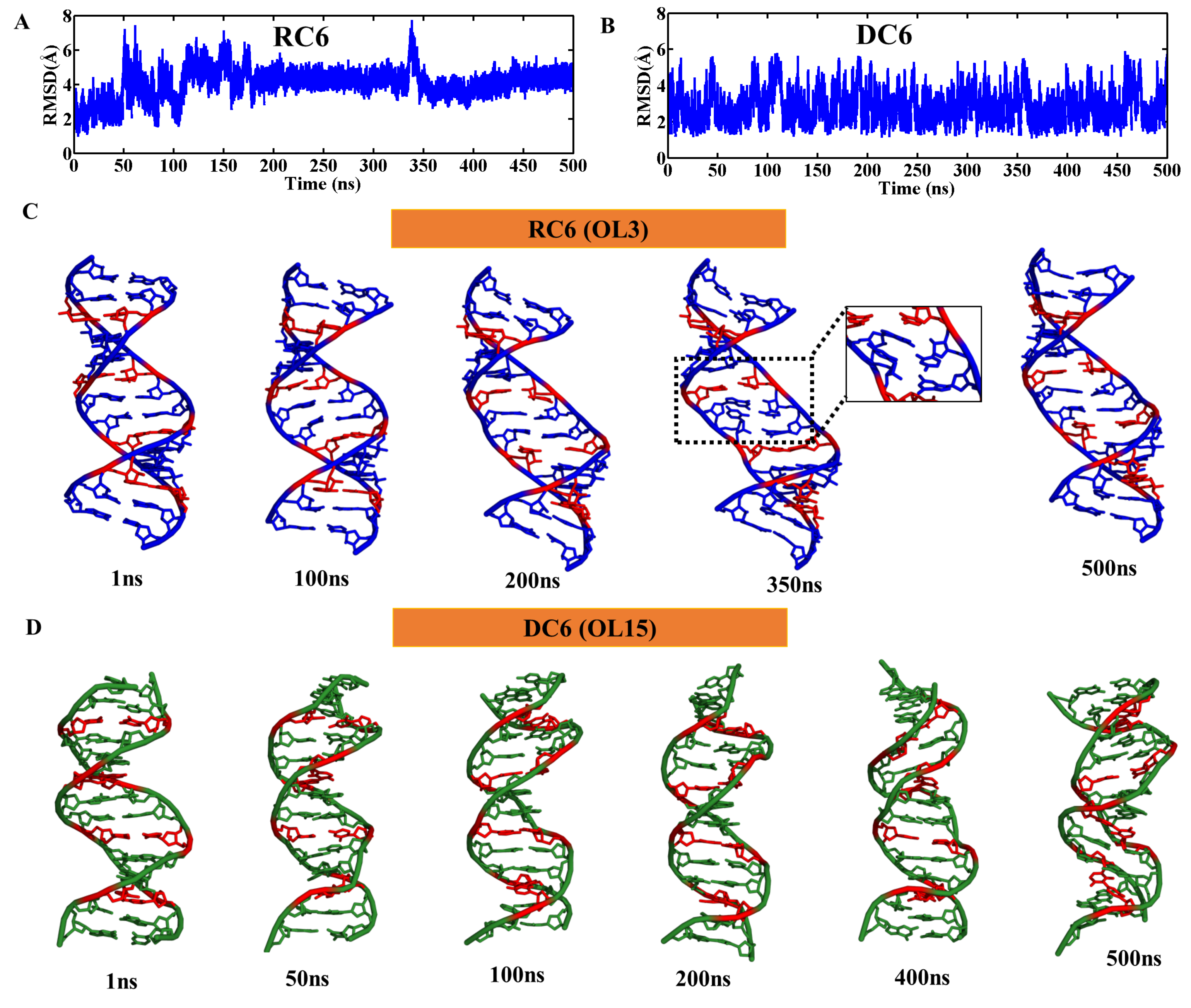 |
| --- |
| **Fig S7.** **Summary of RC6 and DC6 MD simulations carried out using the OL3 & OL15 force fields.** The RMSD value is quite high for RC6 (A) compared with DC6 (B), indicating more distortions induced by the 6 C…C mismatches in the RNA compared with the DNA. A transient structural distortion seen around 350ns (C) is nearly similar to the structural distortions seen in the MD simulations carried out using FF99SB force field (Fig 2B). Note that the C…C mismatches are colored red (C-D). |

| **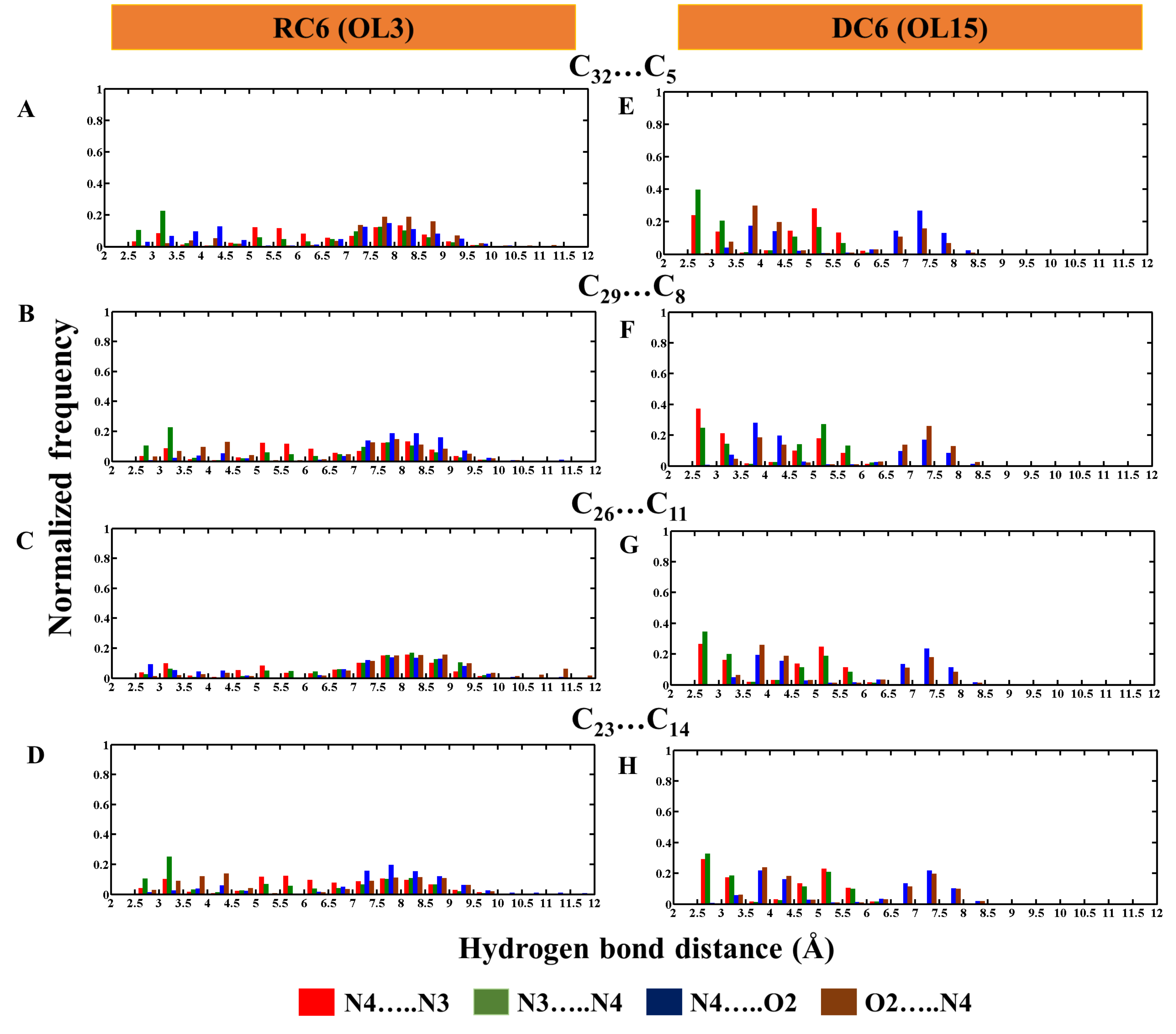** |
| --- |
| **Fig S8. Hydrogen bond distance distribution corresponding to RC6 and DC6.** Normalized frequency of occurrence of hydrogen bond distance for the central 4 C….C mismatches in both RC6 (A-D) and DC6 (E-H) sequences using OL3 and OL15 force fields, respectively. In RC6, the hydrogen bonds are mostly lost, as reflected in fewer populations in the range of 2.5–3.5Å compared with the populations around ~8Å. Such a loss of hydrogen bonds may destabilize the RC6. In contrast, DC6 retains the C…C hydrogen bonds, as can be seen with more populations around 3Å. Note that a terminal CCG triplet on either side of the duplex is excluded for the analysis because of the end-fraying effect. |

| 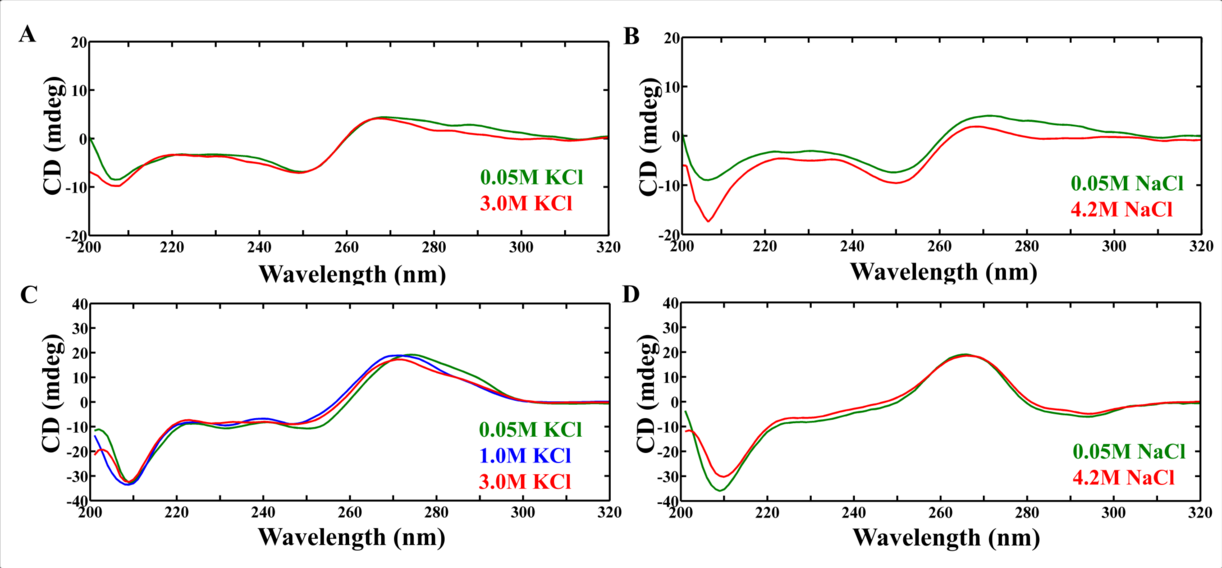 |
| --- |
| **Fig S9.** **Circular dichroism spectra corresponding to the DNA and RNA duplexes consist of canonical base pairs (DWCa & RWCa).** CD spectra indicate the occurrence of duplex conformation in the presence of both KCl and NaCl: (A&B) DNA (DWCa) and C&D) RNA (RWCa). |

| 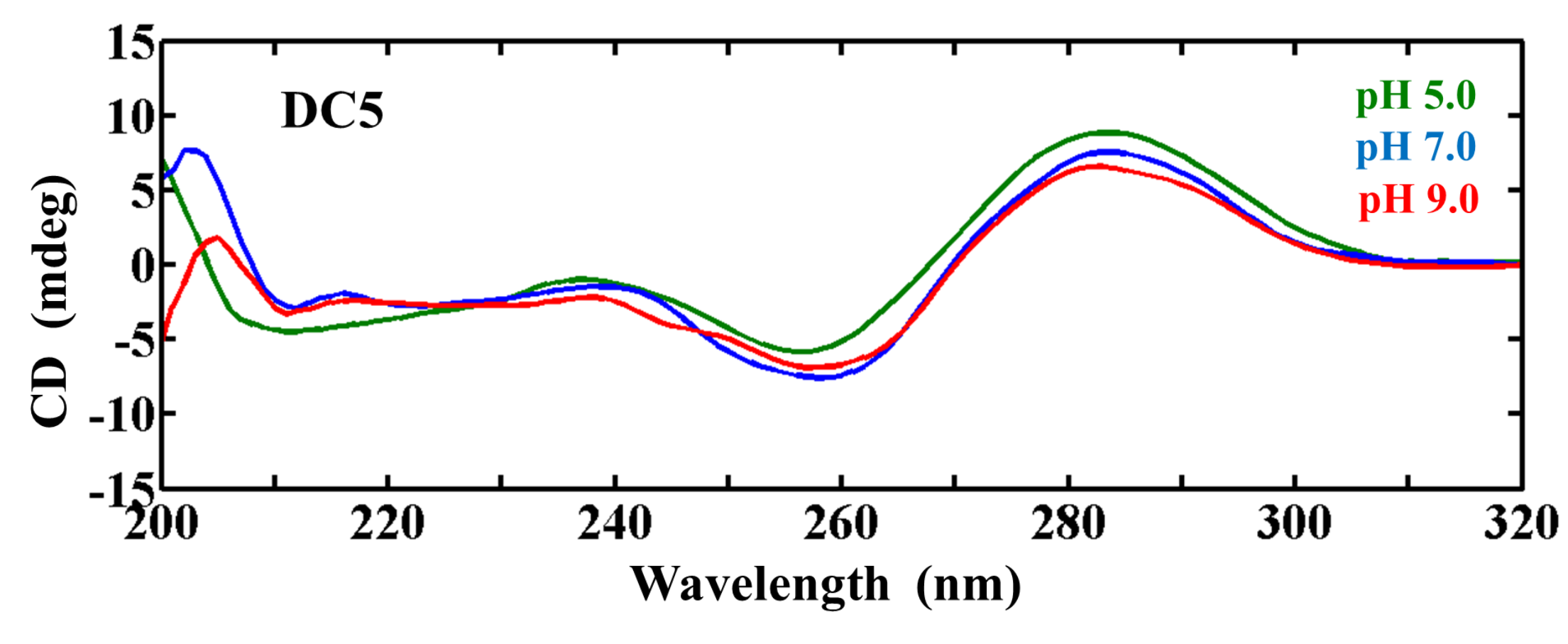 |
| --- |
| **Fig S10.** **CD spectra for DC5 sequence at different pH values.** Irrespective of the pH, DC5 confines to the B-form geometry. |

| 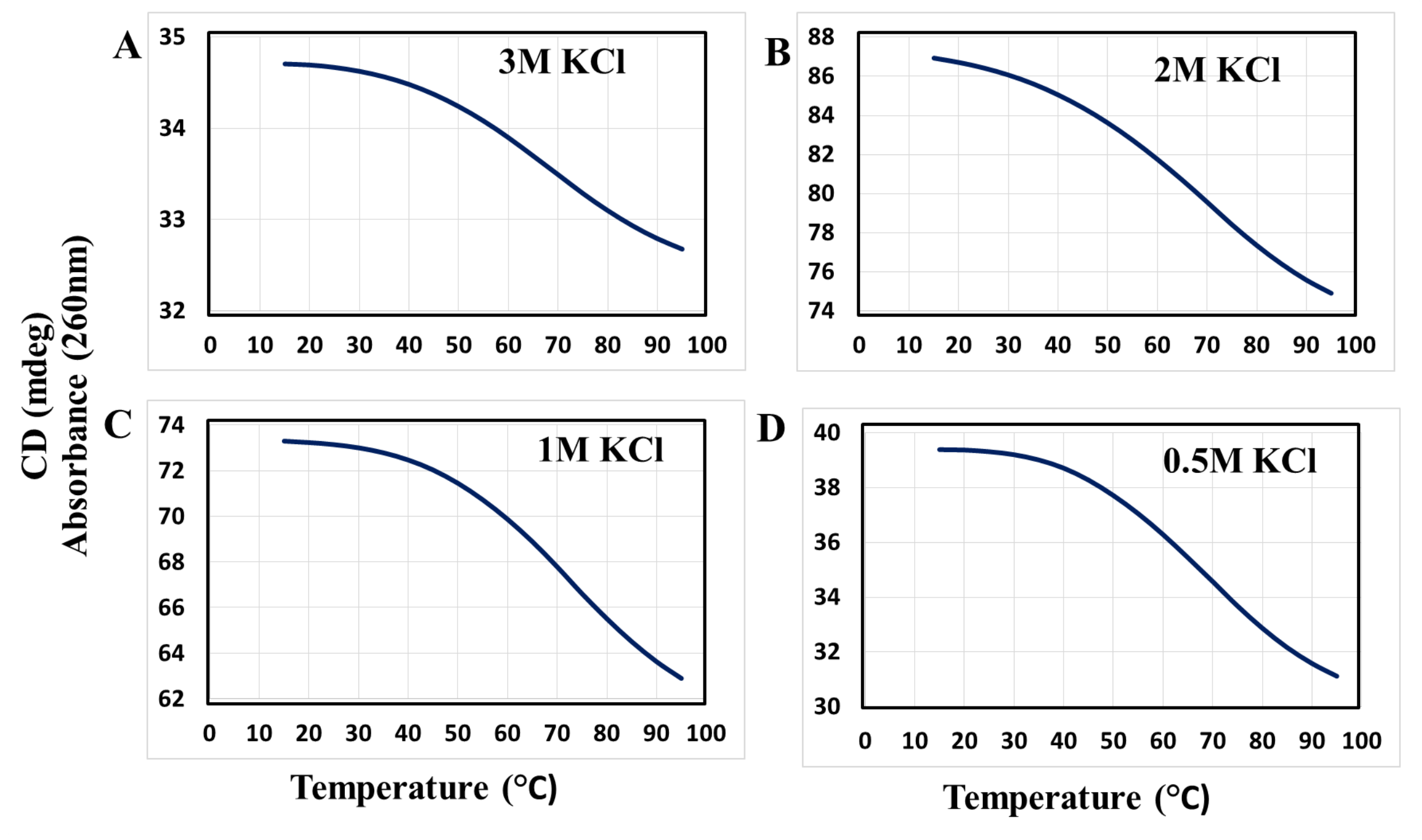 |
| --- |
| **Fig S11. Thermal melt profile for DG15 sequence with high KCl concentrations.** The thermal melting profiles collected at 0.5-3M KCl concentrations for DG15 (A-D) (45mer) confirm the formation of quadruplex. |

| 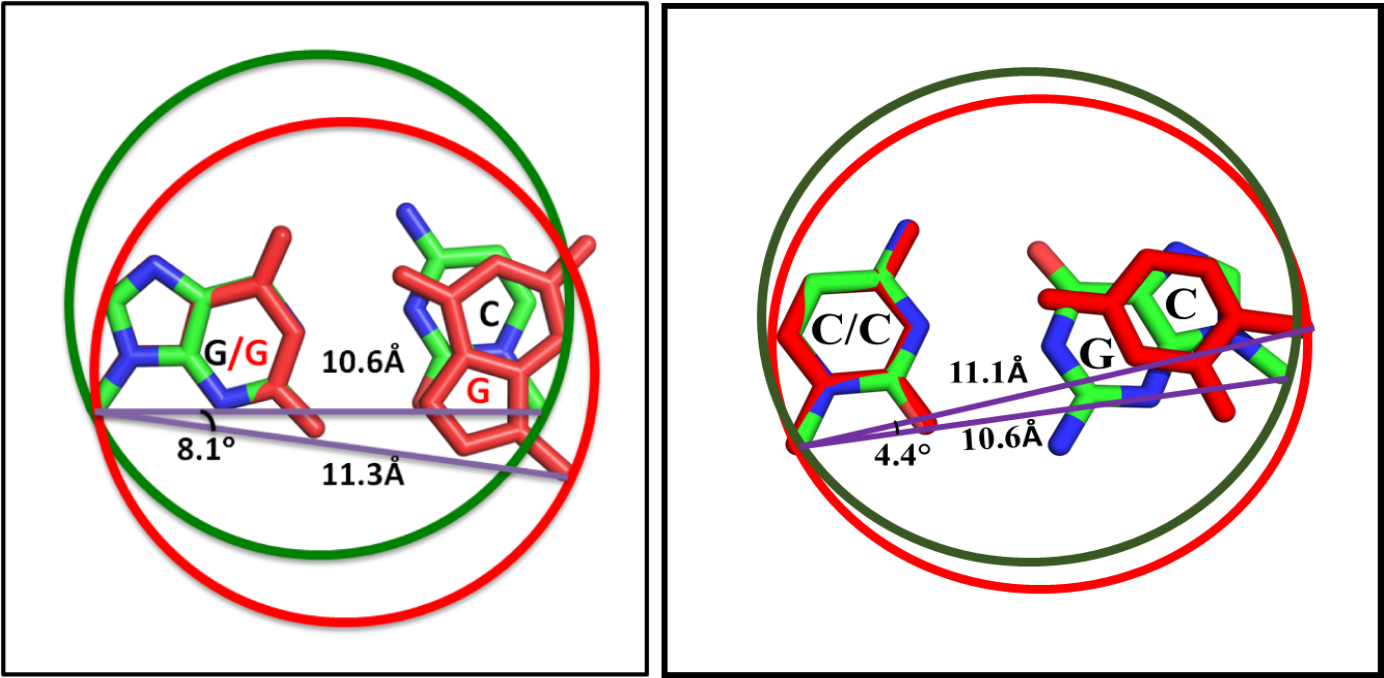 |
| --- |
| **Fig S12. Superposition of the canonical G...C pair with the noncanonical G...G (Left) and C…C (Right) mismatches.** Residual twist and radial difference, the quantitative measures of base pair nonisomorphism, are quite less between G...C and G...G (residual twist = ~8.1° & radial difference = ~0.7Å) and G…C and C…C (residual twist = ~4.4° & radial difference = ~0.5Å) compared with G…C and A…A mismatches (residual twist = ~16° & radial difference = ~1.6Å). Note that the A…A mismatch induces a B-Z junction in the DNA duplex (9, 10). |

| **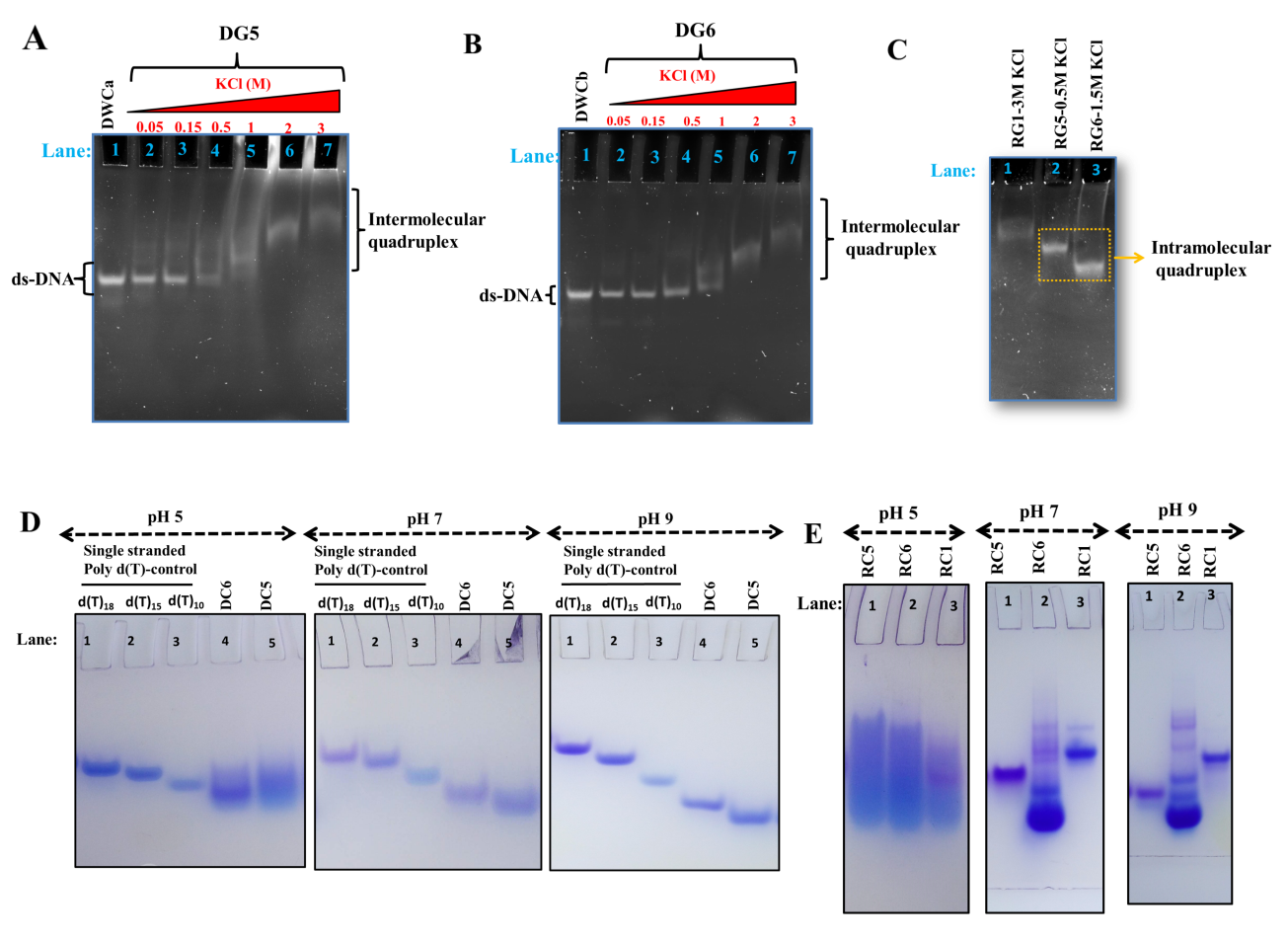** |
| --- |
| **Fig S13. Unprocessed EMSA images of DNA and RNA CGG and CCG sequences given in the main text Figures 1 and 5.** (A-C) EMSA collected with various concentrations of KCl: (A) DG5 (B) DG6 and, (C) RG1 (Left), RG5 (Middle) and RG6 (Right) sequences. (D, E) EMSA collected at pH values 5 (Left), 7 (Middle) and 9 (Right) for the sequences DC6 (D, lane 4), DC5 (D, lane:5), RC5 (E, lane: 1), RC6 (E, lane: 2) and RC1 (E, lane: 3). See Table 1 of the main text for sequence information. |

**References**

1. Perez A, Marchan I, Svozil D, Sponer J, Cheatham TE, 3rd, Laughton CA, et al. Refinement of the AMBER force field for nucleic acids: improving the description of alpha/gamma conformers. Biophys J. 2007;92(11):3817-29.

2. Zgarbova M, Sponer J, Otyepka M, Cheatham TE, 3rd, Galindo-Murillo R, Jurecka P. Refinement of the Sugar-Phosphate Backbone Torsion Beta for AMBER Force Fields Improves the Description of Z- and B-DNA. J Chem Theory Comput. 2015;11(12):5723-36.

3. Zgarbova M, Otyepka M, Sponer J, Mladek A, Banas P, Cheatham TE, 3rd, et al. Refinement of the Cornell et al. Nucleic Acids Force Field Based on Reference Quantum Chemical Calculations of Glycosidic Torsion Profiles. J Chem Theory Comput. 2011;7(9):2886-902.

4. Case D, Betz R, Cerutti DS, Cheatham T, Darden T, Duke R, et al. Amber 16, University of California, San Francisco2016.

5. Patro LPP, Kumar A, Kolimi N, Rathinavelan T. 3D-NuS: A Web Server for Automated Modeling and Visualization of Non-Canonical 3-Dimensional Nucleic Acid Structures. J Mol Biol. 2017;429(16):2438-48.

6. Thenmalarchelvi R, Yathindra N. New insights into DNA triplexes: residual twist and radial difference as measures of base triplet non-isomorphism and their implication to sequence-dependent non-uniform DNA triplex. Nucleic Acids Res. 2005;33(1):43-55.

7. Rathinavelan T, Yathindra N. Base triplet nonisomorphism strongly influences DNA triplex conformation: effect of nonisomorphic G* GC and A* AT triplets and bending of DNA triplexes. Biopolymers. 2006;82(5):443-61.

8. Ananth P, Goldsmith G, Yathindra N. An innate twist between Crick's wobble and Watson-Crick base pairs. RNA. 2013;19(8):1038-53.

9. Khan N, Kolimi N, Rathinavelan T. Twisting right to left: A...A mismatch in a CAG trinucleotide repeat overexpansion provokes left-handed Z-DNA conformation. PLoS Comput Biol. 2015;11(4):e1004162.

10. Kolimi N, Ajjugal Y, Rathinavelan T. A B-Z junction induced by an A ... A mismatch in GAC repeats in the gene for cartilage oligomeric matrix protein promotes binding with the hZalphaADAR1 protein. J Biol Chem. 2017;292(46):18732-46.

11. Mooers BH, Logue JS, Berglund JA. The structural basis of myotonic dystrophy from the crystal structure of CUG repeats. Proc Natl Acad Sci U S A. 2005;102(46):16626-31.
